# Fetal glucocorticoid receptor (*Nr3c1*) deficiency alters the landscape of DNA methylation of murine placenta in a sex-dependent manner and is associated to anxiety-like behavior in adulthood

**DOI:** 10.1038/s41398-018-0348-7

**Published:** 2019-01-17

**Authors:** Michaela Schmidt, Elad Lax, Rudy Zhou, David Cheishvili, Arne Mathias Ruder, Alessia Ludiro, Florian Lapert, Anna Macedo da Cruz, Paolo Sandrini, Teresa Calzoni, Farida Vaisheva, Christiane Brandwein, Alessia Luoni, Renaud Massart, Laurence Lanfumey, Marco Andrea Riva, Michael Deuschle, Peter Gass, Moshe Szyf

**Affiliations:** 1 Central Institute of Mental Health Mannheim (ZI), Medical Faculty of Mannheim, University of Heidelberg, J5, 68159 Mannheim, Germany; 2 Department of Pharmacology & Therapeutics, McGill University, Montreal, QC H3G 1Y6 Canada; 3 Sackler Program for Epigenetics and Psychobiology, McGill University, Montreal, QC H3G 1Y6 Canada; 4 Department of Pharmacological and Biomolecular Sciences, University of Milan, Via Balzaretti, 9, I-20133 Milan, Italy; 5 Inserm, U894, Centre de Psychiatrie et Neurosciences, 75014 Paris, France; 6 Université Paris Descartes, UMRS894, 75014 Paris, France

## Abstract

Prenatal stress defines long-term phenotypes through epigenetic programming of the offspring. These effects are potentially mediated by glucocorticoid release and by sex. We hypothesized that the glucocorticoid receptor (*Gr, Nr3c1*) fashions the DNA methylation profile of offspring. Consistent with this hypothesis, fetal *Nr3c1* heterozygosity leads to altered DNA methylation landscape in fetal placenta in a sex-specific manner. There was a significant overlap of differentially methylated genes in fetal placenta and adult frontal cortex in *Nr3c1* heterozygotes. Phenotypically, *Nr3c1* heterozygotes show significantly more anxiety-like behavior than wildtype. DNA methylation status of fetal placental tissue is significantly correlated with anxiety-like behavior of the same animals in adulthood. Thus, placental DNA methylation might predict behavioral phenotypes in adulthood. Our data supports the hypothesis that *Nr3c1* influences DNA methylation at birth and that DNA methylation in placenta correlates with adult frontal cortex DNA methylation and anxiety-like phenotypes.

## Introduction

The glucocorticoid receptor (GR, NR3C1) plays a major role in the development of stress-induced disorders^1^. Prenatal stress exerts a strong impact on the HPA-axis of rat offspring into adulthood (e.g., ^2,3^). The behavior of affected animals is altered during the whole lifespan: pups display increased ultrasonic vocalizations when stressed prenatally^4^, adolescent rats show less social play^5^, and adult animals show more anxiety- and depressive-like behavior^6^. These results have been found in humans as well^7^. Moreover, there is an abundance of studies showing sex differences in stress-programming regarding behavioral, physiological, endocrine, and epigenetic modifications^8^.

As the stress response is mediated by glucocorticoids, they are potentially a primary programming factor conveying maternal stress to the fetus via the placenta^9^. This assumption is supported by the evidence that treatment with synthetic glucocorticoids of pregnant rodents leads to offspring with similar HPA-axis and behavioral changes as prenatally stressed offspring^10^. Moreover, when maternal adrenal glands are removed, prenatally stressed offspring do not display the full phenotype of increased stress responsivity^11^. There is a large variety of methods for applying prenatal stress in animal models (e.g., ^9,12,13^). Loss of NR3C1 function in the nervous system disturbs hypothalamus–pituitary–adrenal-axis regulation so that glucocorticoid levels^14^ as well as stress responsivity are elevated^15,16^. In this study, we use a heterozygous *Nr3c1* knockout mouse to determine the role of the fetal *Nr3c1* gene in defining DNA methylation patterns and future behavior.

The glucocorticoid receptor has been demonstrated to be a crucial target for epigenetic programming by early postnatal experiences: differences in maternal care in the rat stably alter the methylation state of the promotor region of the glucocorticoid receptor in the hippocampus of offspring^17^. Increased maternal licking/grooming caused a lower stress responsivity of offspring compared to the offspring of dams with low levels of licking/grooming. Epigenetic pharmacological manipulation reversed the effects of maternal care on DNA methylation as well as behavior, supporting the idea that epigenetic differences mediated the effect of maternal care on the behavior of the offspring^17,18^. Since then, these findings have been replicated in other species^19,20^ as well as in humans^21^.

In addition, during fetal development, glucocorticoids play a pivotal role in regulating the placenta that controls fetal exposure to the maternal environment^22^. The placenta expresses *11b-hydroxysteroid dehydrogenase type-2* (*11b-HSD2*), which protects the fetus from excessive maternal glucocorticoids by metabolizing corticosterone into inactive 11-dehydrocorticosterone^23^. However, a significant reduction of this enzyme and its placental gene expression after repeated stress exposure in pregnancy has been observed^24^, so that it is not able to completely shield the fetus from glucocorticoid overexposure caused by the maternal stress reaction.

Crudo et al.^25,26^ reported global methylation changes in placental tissue and organs of the fetus after maternal betamethasone treatment in guinea-pigs. Global methylation remained altered in adult tissues of the offspring. Furthermore, the maternal betamethasone treatment also modifies DNA methylation and histone H3 lysine 9 acetylation in the fetal hippocampus^27^ and leads to differential *Gr* binding to a large number of different gene promotors and methylation of specific GREs in the fetal hippocampal *Mr* gene^26^.

We hypothesized that *Nr3c1* plays an important role in mediating the effects of stress on DNA methylation patterns systemically during the development and that these alterations mediate phenotypic effects of altered stress and glucocorticoid levels. We also reasoned that these methylation changes occur across several tissues and are not exclusive to the brain. We focused on fetal placenta since it is one of the few noninvasive sources of early life biological material at birth beside blood^28,29^ and perhaps one of the few biological sources that enable correlating DNA methylation at birth and later phenotypes in the same living animal.

As placental tissue is usually consumed by the mouse dam soon after birth, it is impossible to collect it in a natural setting. There are few studies linking characteristics of placenta and behavior of offspring in adulthood and they usually assign different litters to sample tissues and the behavioral tests (e.g., ^30,31^).

We succeeded in establishing an elaborate procedure of caesarian section^32^ that allowed the pups to develop with minimal neurotoxic side effects and provided us with placental tissue from the same individuals. Due to an upbringing with foster dams, the animals developed into adulthood and were tested for behavioral phenotypes as adults. This allowed us to measure in the same animal the baseline DNA methylation pattern in placenta and behavioral phenotypes later in life. To our knowledge, the present study is the first one linking epigenetic features of fetal placental tissue directly to the behavior of the same animals in adulthood. Our data suggest broad and physiologically pertinent changes in DNA methylation in placenta that correlate with anxiety-like behavior in adulthood in response to *Nr3c1* deficiency.

## Methods

Full details of all methods can be found in the supplementary file.

All procedures complied with the regulations covering animal experimentation within the EU (European Communities Council Directive 86/609/EEC) as well as national and local authorities (Regierungspräsidium Karlsruhe, Germany).

### Animals and tissue collection

Acclimatized female *Nr3c1*^+/+^ and male *Nr3c1*^+/−^ mice were used for breeding. *Nr3c1*^+/−^ animals were originally generated as described by Tronche et al.^14,33^. Pregnant females delivered by caesarean sections on E18.5 post conception. The procedure was completed within 10–20 min (11.5 min in average) using xenon gas and isoflurane anesthesia in order to protect the pups’ brain and heart from hypoxia-induced damage^32^. The fetal placental tissue was kept on dry ice and stored at −80 °C immediately after dissection. After caesarean sections, the biological dams were sacrificed and experienced C57BL/6N foster dams whose own litter was aged between PND (postnatal day) 1 and PND 4 were used to raise the pups. The transfer into the nest of the lactating foster dam took place within 15 min after birth. The original pups were mixed with litter and urine of the foster dam to pick up her scent and so improving acceptance by the foster dam. We pooled the samples of individual placentae for the genome-wide epigenetic analysis to 3 groups of placental tissue per sex/genotype combination (Table S1).

Adult frontal cortex was collected from a different cohort of male GR-i animals (according to ref. ^34^). These animals expressed GR antisense mRNA and consequently exhibited a decrease in GR-specific binding as well as GR mRNA levels in the frontal cortex^35^. Eight samples of frontal cortex were pooled and analyzed via ChIP-bisulfite-Seq (for a more detailed description please see supplementary methods).

### Behavior

Behavioral tests (Novel Cage, Open Field, Forced Swim Test, Hot Plate Test, Learned Helplessness and Dark Light Box as described elsewhere^10,36^) were performed by a completely blinded investigator. Maternal care was monitored from PND2 to PND8 for 24 h a day and rated via instantaneous sampling with a detailed ethogram adapted by Coutellier et al.^37^. Maternal care did not significantly differ between the dams (see Table S2 and Table S3).

### Statistics

All analyses of variance (ANOVA) were conducted using the general linear model (GLM). Whenever the assumptions of parametric analysis were not met by graphical examination of homoscedasticity, raw data were transformed according to the Box–Cox-method or an outlier analysis was performed. All analyses of variance were based on a 2 × 2 factorial design with factors “sex” and “genotype”. Two-way univariate ANOVAs were performed for weight of adrenals, spleens, birth weight, length of the newborn, rearing of novel cage, hot plate, sub-tests of dark–light-box, FST, learned helplessness, and methylation validation with pyrosequencing. For open field test and body weights over time, a two-way repeated measurements ANOVA was conducted with the factor “time”. Linear Pearson product-moment coefficient was applied to determine correlations between DNA methylation and behavioral measures. Spearman rho coefficient was used for correlating number of failures and escape latency in the learned helplessness paradigm. Moderation effects were tested using Hayes Process macro for SPSS^38^. A MANOVA with subsequent univariate two-way ANOVAs and *t*-tests were performed for the analysis of global methylation levels. For DNA methylation analysis and annotation, the R package “methylkit” was used^39^, correcting for multiple testing using Benjamini–Hochberg false discovery rate (FDR). A non-parametric *χ*^2^-test was calculated to detect differences between all 19 foster dams in maternal care behavior. The hypergeometric test as well as the binomial test was applied to test significant overlaps. Adjusted *p*-value ≤ 0.05 was set as threshold for statistical significance except for genome wide DNA methylation data which was set at 0.2. Statistical analyses were performed using SPSS 21.0 software package for Windows.

### Capture bisulfite sequencing and DNA methylation mapping

SeqCap Epi Enrichment System (Roche-NimbleGen) performed at the Institute de recherches cliniques de Montréal was used for targeted bisulfite sequencing of promoters and enhancers.

### Analysis of differentially methylated cytosines

FastQC assessed sequencing scores and other quality metrics. Methylation levels and coverage levels were extracted with methratio.py command in Bsmap. Differential methylation cytosines were analyzed with methylKit R package^39^ with FDR threshold of 0.2. Differentially methylated positions were annotated with HOMER^40^.

## Results

### Effects of *Nr3c1*^+/−^ deficiency on behavior

#### *Nr3c1*^+/−^ mice display an increase in anxiety-like behavior as measured by the dark–light box

**Fig. 1:**
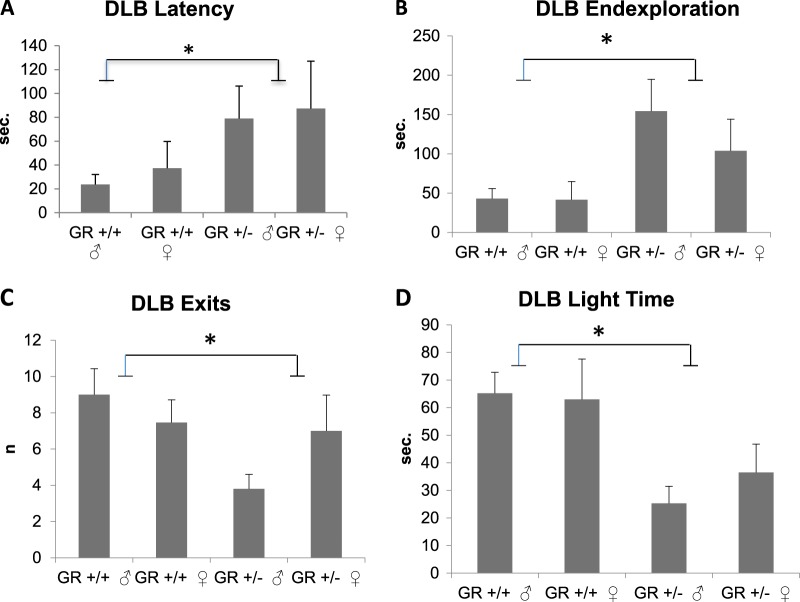
Anxiety-like behavior measured by the dark–light-box. **a** Increased latency to enter the light department in *Gr (Nr3c1)*^+/−^ animals (*p* = 0.011, *F*_3,44_ = 7.047). **b** Increased latency to explore the end of the compartment in *Gr (Nr3c1)*^*+/−*^ animals (*p* = 0.008, *F*_3,44_ = 7.798). **c** Significant decrease in the number of exits in *Gr (Nr3c1)*^+/−^ animals (*p* = 0.013, *F*_3,44_ = 6.646). **d** Decreased duration of time spent in the light area in *Gr (Nr3c1)*^+/−^ animals (*p* = 0.001, *F*_3,44_ = 14.059). Means ± SEM

*Nr3c1* depletion significantly increased anxiety-like behavior in all parameters monitored in the dark–light box in both sexes (Fig. 1a–d). Nr3c1 animals spent less time in the light compartment which is considered a measure of anxiety-like behavior in rodents. Maternal care, novel cage test, locomotor activity, depressive-like behaviors such as forced swim test and learned helplessness, body weight, adrenal and spleen size were not affected by *Nr3c1* deficiency (please see supplementary results).

#### *Nr3c1* deficiency affects DNA methylation in a sex-dependent manner

**Fig. 2:**
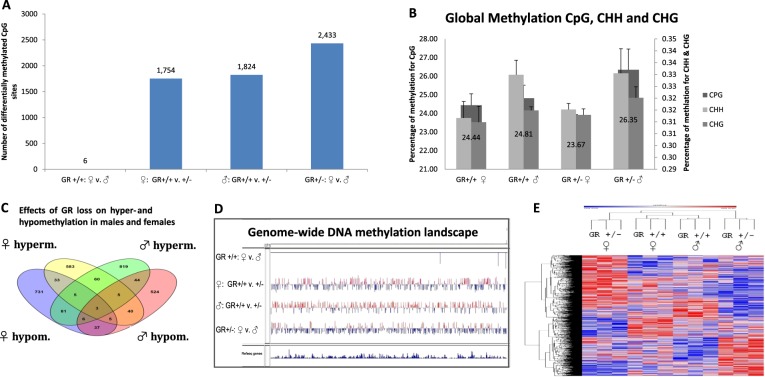
Sex-dependent effects of *Nr3c1* deficiency. **a** Number of differentially methylated CpG sites for the comparisons of female vs male *Nr3c1*^+/+^, female *Nr3c1*^+/+^ vs female *Nr3c1*^+/−^, male *Nr3c1*^+/+^ vs *Nr3c1*^+/−^, and female vs male *Nr3c1*^+/−^ as depicted in the *x*-axis. Numbers directly above the graph bars represent absolute numbers of differentially methylated CpG sites. **b** Global methylation of CpG, CHH, and CHG sites in wildtype and *Nr3c1* knockouts in both sexes. Numbers depicted directly on the graphs represent the percentage of CpG sites (see also *y*-axis to the left). The percentage of CHH and CHG sites are illustrated on the right *y*-axis. **c** Effects of *Nr3c1* loss on hyper- and hypomethylation in males and females. **d** Genome-wide methylation tracks for CpG sites using Integrative Genomics Viewer (Broad Institute). Comparison is shown for female vs male wildtype, female wild type vs female *Nr3c1* heterozygous, male wild type vs male *Nr3c1* heterozygous, and male vs female *Nr3c1* heterozygous animals. **e** DNA methylation landscape of *Nr3c1 deficiency* in male and female placental DNA. Heatmap (row distance metric: Pearson correlation, average linkage) depicting the clustering of 6017 CpGs that were differentially methylated (*q* < 0.2) between placentae of *Gr*^+/+^ and *Gr*^+/−^ fetuses of both sexes. Rows correspond to CpGs and columns to animals’ genotype and sex. Here, group number 1–3 indicates three pooled samples of *Gr*^+/+^ males, number 4–6 stands for three pooled samples of *Gr*^+/−^ males, 19–21 for three pooled samples of *Gr*^+/+^ females, and 22–24 for three pooled samples of *Gr*^+/−^ females. Red indicates higher methylation in a row and blue indicates lower methylation. See also Table S4-S9

The genome wide state of methylation of promoters and enhancers was mapped using capture bisulfite sequencing (Fig. 2) and validated by pyro-sequencing (Fig. S1). In contrast to wild type animals which exhibit a small number of different methylation sites between sexes^6^ (see Fig. 2a, d), 2433 differentially methylated CpG sites were noted between males and females in *Nr3c1* depleted animals (Table S4).

There was a multivariate interaction effect of sex*genotype on global methylation of cytosines at CpG, CHH, and CHG contexts (*p* = 0.013). Sex was significant for CHH (*p* = 0.045, *F*_3,8_ = 5.647) and almost reached significance for CpG (*p* = 0.072, *F*_3,8_ = 4.276). Stratified for genotype, there was a statistical tendency for sex only in *Nr3c1*^*+/−*^ animals (*p* = 0.079, *t*_4_ = 2.341) with males showing higher global methylation levels (see Fig. 2b).

Hypermethylated sites were more abundant in male *Nr3c1* deficient animals (1023 in males vs 734 in females (*p* = 2.869E−12, binomial test), see Fig. 2c, d and Fig. S2) while hypomethylated sites were more frequent in females (901 in females vs 664 in males (*p* = 1.139E−09, binomial test)). There was however a significant overlap between sites that are hypermethylated (hypergeometric test *p* = 4.983E−11) or hypomethylated (hypergeometric test *p* = 2.435E−06) in both males and females. Each one of the four groups clusters separately (see Fig. 2e).

*Nr3c1*^+/−^ deficiency causes significant loss of methylation in 51 genes and gain of methylation in 73 genes in both sexes (annotation: 15 reads, delta beta = 0.2, FDR < 0.2); 95 genes were hypermethylated in males and hypomethylated in females while 53 genes were hypomethylated in males and hypermethylated in females (Table S5–8).

Significant sex-dependent methylation in the opposite direction at the same CG site (hypermethylation in males and hypomethylation in females and vice versa) was detected in 10 sites: ***Gm16853***, ***Mir6998***, ***Rgs22***, ***Coro2b***, ***Babam1***, ***Hoxd9***, ***Gmppa***, ***Slc14a2***, ***Lhcgr***, ***Cacnb1***. Seven CGs were differentially methylated in *Nr3c1*^+/−^ in the same direction in males and females: ***Paqr4***, ***4930487D11Rik***, ***Rusc1***, ***Fshr***, ***5033406O09Rik***, ***Sumo2***, ***Mir7078*** (see Table S9).

### Effects of *Nr3c1* heterozygosity and sex on methylation state of candidate genes

We then examined methylation in a shortlist of genes that were previously associated with early life stress and psychiatric disorders as well as placental functioning: *Ank3, Avp, Avpr1a, Avpr1b, Bdnf, Cacna1c, Cyp11b1, Cyp11b2, Fkbp5, Hsd11b1, Igf2, Morc1, Nr3c1, Oxt, Oxtr, Pclo, Slc6a4*.

There is no main or interaction effect of *Nr3c1* genotype on methylation of *Nr3c1*. For *Igf2*, there is a significant main effect of sex on methylation (*p* < 0.001, *F*_3,8_ = 25.378) with lower methylation in females. A significant sex*genotype-interaction was found for *Hsd11b1* (*F*_3,8_ = 32.883, *p* < 0.001) and *Cacna1c* (*F*_3,8_ = 5,689; *p* = 0.044) but no main effect of either sex or genotype. However, *Nr3c1* deficiency significantly affected methylation of the proximal GC regulator *Fkbp5* which is significantly hypomethylated in both male and female *Nr3c1*^*+/*−^ mice (*F*_3,8_ = 10.537, *p* = 0.012) (see Table S10).

### Functional analysis of sex-dependent alteration of methylation induced by *Nr3c1* deficiency

**Fig. 3:**
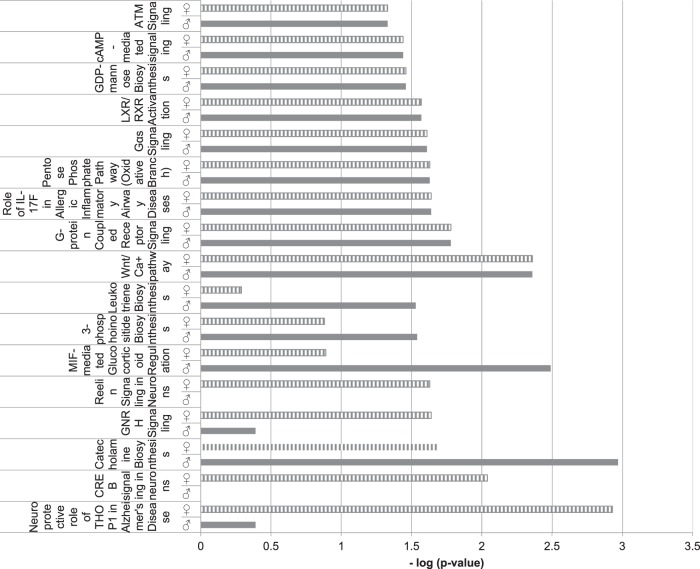
Ingenuity pathway analysis. Selected canonical pathways for the comparison of methylation changes in males and females after *Nr3c1* loss. See also Table S11 and S12

We analyzed potential canonical pathways using ingenuity pathway analysis: a difference between males and females in the level of enrichment of DNA methylation alterations in *Nr3c1* deficient mice was shown in central nervous system-related functions like Reelin and CREB signaling in neurons, whereas ubiquitous cell signaling pathways like the Wnt/Ca+ pathway coupled receptor were found to be similarly affected in both sexes (Fig. 3).

Beside cell signaling and developmental functions many molecules regulating neuronal and metabolic processes were associated with differentially methylated genes in both sexes (see Table S11) and the top upstream regulator was the central nervous system-specific gene *Cannabinoid receptor 1 (CNR1)* (see Table S12).

Five pathways directly related to central neurotransmission were enriched in genes that were hypermethylated in males and hypomethylated in females, whereas the pathways enriched for genes that were hypomethylated in males and hypermethylated in females included mainly hormonal and general cell signaling pathways. There are only two common canonical pathways for the 95 genes hypermethylated in males and at the same time hypomethylated in females and the 53 genes that were hypomethylated in males and hypermethylated in females: G-Protein Coupled Receptor Signaling and Gαi signaling (see Fig. S3 and S4).

### Overlap in differentially methylated promoters in *Nr3c1*-deficient mice in fetal placenta and adult frontal cortex

We examined whether there is an overlap between the genes differentially methylated in placenta of *Nr3c1*^+/−^ male fetuses (based on capture sequencing analysis of the present study) and frontal cortex of adult male Gr-I mice from a separate study (unpublished, based on a ChIP-bisulfite-Seq-analysis of GR-bound DNA methylation). 242 genes were differentially hyper- and hypomethylated in both tissues (17.5% of the genes that are differentially methylated in male fetal *Nr3c1*^+/−^ placentae) (*p* = 0.000388, hypergeometric test, FDR < 0.2; see Table S13).

**Fig. 4:**
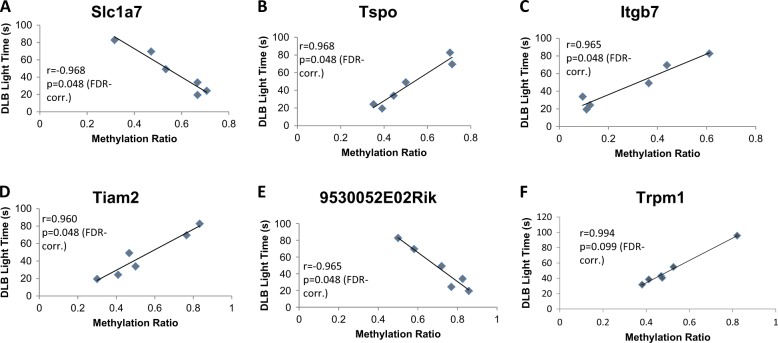
Pearson correlation between methylation ratio and anxiety as measured by the time spent in the light department of the dark–light-box. **a–e** Depicted are line charts for the genes *Slc1a7, Tspo, Itgb7, Tiam2*, and 9530052E02Rik of male animals. **f**Correlation between time spent in the light and *Trpm1* methylation in female animals. All values are FDR-corrected *p-values*

Among others, specific neurotransmitter systems like serotonin and glutamate receptor signaling as well as neuronal developmental (e.g., netrin signaling) and neuroprotective (TNFR2-signaling) pathways were found enriched (see Fig. 4).

Brain functions as well as metabolic-related functions were enriched in both tissues. Additionally, molecules known to be involved in fundamental central nervous functions (see Table S14), molecules involved in basal metabolic processes and oxidative stress as well as a considerable number of microRNA loci (see Table S15) were enriched with differentially methylated genes in placenta as well as frontal cortex. For both tissues, the transcription factors *Fos* and *Atn1* were found as upstream regulators (see Table S16).

### Correlation between differentially methylated genes and anxiety-like behavior in *Nr3c1* deficient mice

**Fig. 5:**
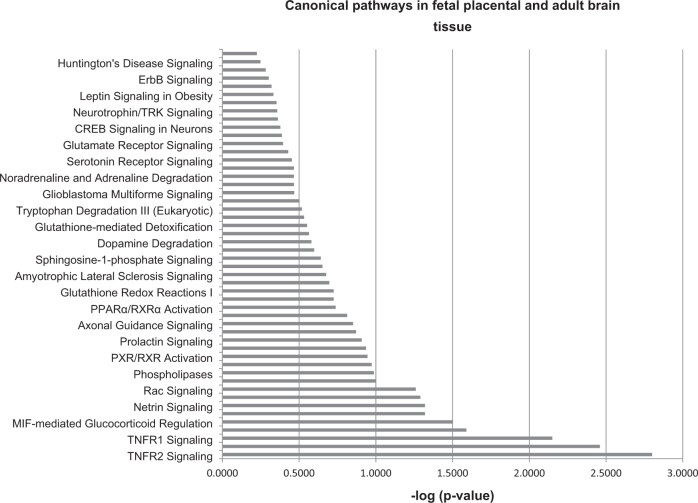
Ingenuity pathway analysis of genes that are differentially methylated in frontal cortex from Nr3c1 antisense animals and placentae from *Nr3c1* heterozygotes. Selected canonical pathways that are differentially methylated in *NR3C1* ^+ /−^ mice in fetal placental and adult Nr3c1 antisense mice frontal cortex. See also Table S13-S15

We correlated the quantitative levels of methylation of CG sites of genes commonly differentially methylated in both sexes (see Table S17) with quantitative behavioral scores for each individual mouse. The behavioral score of anxiety-like behavior of the adult males—but not females—yielded 5 significant correlations with *Slc1a7*, *9530052E02Rik*, *Itgb7*, *Tiam2*, and *Tspo* (see Fig. 5a–f, adjusted *p* = 0.048).

The correlation of methylation levels with depression as measured by the Forced Swim Test and Learned Helpless Paradigm as well as locomotor activity (Novel Cage and Open Field Test) did not lead to significant results consistent with the lack of impact of Nr3c1 heterozygosity on the latter behaviors (please see supplementary results).

### Moderation of levels of anxiety-like behavior in adulthood by differential DNA methylation in fetal placenta

Finally, we assessed the moderating influence of candidate genes whose level of methylation is associated with the *Nr3c1* genotype on anxiety-like behavior: *Ank3, Avp, Avpr1a, Avpr1b, Bdnf, Cacna1c, Cyp11b1, Cyp11b2, Fkbp5, Hsd11b1, Igf2, Morc1, Nr3c1, Oxt, Oxtr, Pclo, Slc6a4*. Methylation of *Fkbp5* significantly moderated *Nr3c1* influence on anxiety-like behavior in adulthood (*F*_1,8_ = 18.1987, *p* = 0.0027). The higher *Fkbp5*’s methylation level, the longer the animals spent in the light indicating a lower level of anxiety-like behavior. The average methylation of *Fkbp5* was significantly reduced in both male and female *Nr3c1* depleted animals (delta for males = −0.254, females = −0.341, *F*_3,8_ = 10.537, *p* = 0.012, see Table S10).

## Discussion

Our study tests for the first time the hypothesis that *Nr3c1* plays a causal role in shaping the DNA methylation profile of the fetus and that these changes in methylation triggered by *Nr3c1* deficiency at birth are associated with phenotypic alterations. *Nr3c1*^*+/−*^ animals of both sexes showed a significant increase exclusively in anxiety-like behavior which is consistent with data derived from several different transgenic mouse models^41,42^. Two of the five genes whose methylation state in placenta significantly correlate with levels of anxiety-like behavior only in adult males but not females: *Tspo* and *Slc1a7* are involved in central nervous system functions. *Tspo* polymorphism is linked to separation anxiety in depressive patients^43^ and *Slc1a7* inhibits glutamate receptor activity^44^.

*Nr3c1* deficiency dramatically enhances differences in DNA methylation between the sexes. This provides a possible mechanism for sex-dependent effects of prenatal stress that were previously reported (e.g., ^45–48^). Although we examined DNA methylation in the placenta, which reflects prenatal experience, it should be noted that early postnatal stress also leads to sex-specific effects later in life and the role of *Nr3c1* in these effects needs further study (e.g., ^49–54^). In addition, it should be noted that perinatal gonadal hormones are capable of inducing changes in methylation patterns between the sexes in wild type animals as well^55,56^.

Nugent et al.^57^ examined the highly sexually dimorphic preoptic area of the rat hypothalamus. They report reduced activity of DNA methyltransferase in this as well as decreased DNA methylation and release of masculinizing genes from epigenetic repression. Gonadal steroids are physiologically released in the first days after birth. However, the sex differences in the fetal part of placenta of the present study were detected on day 18.5 post-conception well before the influence of the postnatal testosterone and its metabolite estradiol. Furthermore, Nugent et al.^57^ describe an astonishingly small number of differentially methylated genes between the sexes exactly as reported in our study: there were only 6 sites differentially methylated between males and females of our wildtype control group vs 2433 sites between our *Nr3c1*-heterozygous female and male animals. The fact that the differences in methylation between the sexes were dramatically enhanced in the heterozygous animals indicates a gene by sex effect on DNA methylation.

Furthermore, Nugent et al.^57^ found higher levels of global CpG-methylation in females in the preoptic area of the hypothalamus. This finding is different from our observation of elevated CpG-methylation in the male placenta of *Nr3c1*^+/−^ animals. Since methylation is highly tissue-specific, this might explain the observed differences in global methylation between the two studies. However, it is possible that *Nr3c1* deficiency changed the DNA methylation landscape independently of postnatal hormonal influence and switched the differences in global methylation between the sexes.

In addition, we examined the effect of sex and *Nr3c1* deficiency on selected candidate genes that are known to be involved in responses to prenatal stress. The growth promoting hormone *Igf2* was significantly less methylated in wild type females as compared to males. Mina et al.^58^ previously reported elevated *Igf2* mRNA in fetal female as compared to male placenta of distressed mothers. Although we observed a sex effect on *Igf2* DNA methylation, there was no effect of *Nr3c1* deficiency. However, *Hsd11b1*, encoding a protein which converts cortisol to the inactive metabolite cortisone is hypermethylated in *Nr3c1*^*+/−*^ males as compared to *Nr3c1*^*+/*−^ females and to wildtype males. Although Green et al.^59^ did not find a sex difference in *Hsd11b1* methylation in human placenta, low methylation of *Hsd11b1* was associated with the risk of being born large for gestational age. Similarly, a sex*genotype interaction effect on DNA methylation was found for *Slc6a4* and *Cacna1* which were previously associated with stress-related disorders^60^.

*Fkbp5*, the proximal regulator of the glucocorticoid receptor, was hypomethylated in placental tissue of our *Nr3c1*^*+/*−^ animals. This result is in line with findings of St-Cyr et al.^61^ who report hypomethylation of *Fkpb5* in the amygdala of adult female offspring as well as increased ACTH stress reactivity after exposure to the prenatal odor of a predator. *Fkbp5* polymorphisms enhance the risk of developing stress-related disorders in adulthood after early traumatic experiences^62^. This effect is allele-specific and depends on epigenetic changes in the glucocorticoid response elements of *Fkbp5*^63^. Most interestingly, we also found a significant moderation effect of *Fkbp5*’s CG-methylation level on anxiety-like behavior in adulthood. Hartmann et al.^64^ succeeded in reducing an anxious-like phenotype caused by an overexpression of *FKBP51* in the basolateral amygdala by applying a highly selective *FKBP51* point mutation antagonist. A new specific antagonist of FKBP5—SAFIT2—reduced anxiety-like behavior even when administered peripherally. It would be intriguing to test if SAFIT2 could also rescue the anxious-like phenotype of our *Nr3c1*^*+/−*^ animals.

Besides hormonal receptors and microRNAs, several genes that are differentially methylated in both sexes in the same CGs when *Nr3c1* is deficient are also associated with neuronal and mental disorders (6 out of 17 CGs). Four of these genes were differentially methylated in the opposite direction: *Babam1* has been linked to schizophrenia^65^, *Cacnb1* to autism^66^ and depression^67^, *Coro2b*, a candidate gene for ciliopathies to intellectual disability^68^ and *Gmppa* to intellectual disability and autonomic dysfunction^69^. Two genes that are differentially methylated in the same direction in males and females are *Rusc1* whose gene product serves as neuronal adaptor protein^70^ and *Paqr4* which was recently associated with the development of epilepsy^71^.

The functional pathway analysis revealed that the top upstream regulator of differentially methylated genes in both sexes in response to *Nr3c1* deficiency is the *Cannabinoid receptor 1* (*CNR1*) gene which is expressed in the central nervous system and is known to be associated with exploratory drive, anxiety and stress response^72^. Most interestingly, the endocannabinoid receptor is also discussed as a possible target for anxiolytic drugs^73^.

To our knowledge, there is only one study that examined the correlation of gene expression in response to prenatal stress in placenta with adult behavior: Mueller and Bale^31^ found a maladaptive stress responsivity in adulthood in males exposed to prenatal stress, which was linked to a significantly increased gene expression of *PPARα*, *IGFBP-1*, *HIF3α*, and *GLUT4* in male placenta. The authors propose a mechanism by which the expression of *PPARα* is increased by glucocorticoids^74^, which in turn induces expression of IGFBP-1^75^. Interestingly, *PPARγ* and *IGFBP-2* are differentially methylated in frontal cortex as well as placenta of *Nr3c1* deficient males of our study.

PPARγ-agonists that are usually used for treating type-2 diabetes have been reported to exhibit antidepressant effects as well^76^. *IGFBP-2* has also been linked to diabetes and depression in peripheral^77^ and central nervous tissue^78^. This illustrates the potential overlap between psychiatric disorders and metabolic dysfunctions as reported before^79^.

Our data point to the potential utility of placental DNA methylation markers for early diagnosis of prenatal stress and for prediction of the emergence of behavioral disorders later in life. Thus, further evaluation of placental tissue in mice as well as humans, which is easily available after delivery, as a possible source for the predictor of risk for adult psychiatric disorders at birth seems very promising.

## Supplementary information

Supplemental Methods clean version

Supplemental Figures

Supplemental Tables

Supplemental Results

Supplemental legends

## Supplementary information

**Supplementary Information** accompanies this paper at (10.1038/s41398-018-0348-7). Raw data are archived in the Gene Expression Omnibus under the following accession numbers: GSE123188 and GSE123460.
