# Supplementary material for: Fetal glucocorticoid receptor (Nr3c1) deficiency alters the landscape of DNA methylation of murine placenta in a sex-dependent manner and is associated to anxiety-like behavior in adulthood
Source: Transl Psychiatry. 2019 Jan 17;9:23. doi: 10.1038/s41398-018-0348-7 (PMC6336883; doi:10.1038/s41398-018-0348-7)
Supplement: Supplementary file 1 — Supplemental Methods clean version [file 41398_2018_348_MOESM1_ESM.docx]

**Supplementary methods**

**Animals and tissue collection**

Acclimatized female *Nr3c1^+/+^* and male *Nr3c1^+/^*^-^ mice were used for breeding. *Nr3c1^+/^*^-^ animals were originally generated as described by Tronche, Kellendonk (1) and Tronche, Kellendonk (2). Pregnant females delivered by caesarean sections on E18.5 p.c. The procedure was completed within 10–20 min (11.5 min in average) using xenon gas and isoflurane anesthesia in order to protect the pups’ brain and heart from hypoxia-induced damage (3). The fetal placental tissue was kept on dry ice and stored at -80^0^C immediately after dissection. After caesarean sections, the biological dams were sacrificed and experienced C57BL/6N foster dams whose own litter was aged between PND (postnatal day) 1 – PND 4 were used to raise the pups.

15 male and female offspring per genotype were housed individually after weaning from four weeks of age in macrolon type II cages with nesting material under a reversed day-night cycle (lights on from 19.00-07:00 hrs with 12h dark and 12h light phase) and supplied with food and water ad libitum. Only one heterozygous or wildtype male or female respectively was used per litter to prevent litter effects (4).

Brains of adult C57BL/6N male *Nr3c1^++^ (GR^++^)* and GR-I mice of another cohort according to (5) were removed after sacrificing the animals. Frontal cortices were rapidly dissected, kept on dry ice and stored at -80^0^C afterwards.

**Body, adrenal and spleen weights**

Body weights of offspring were taken at birth and at 4, 8, 12, 16, 20 weeks of age. All animals were sacrificed 2-3 weeks after the last experiment at the age of 21-25 weeks by decapitation between 8.00 and 11.00 AM. Adrenals and spleens were dissected, weighed and frozen on dry ice at -80 degrees.

**Extraction of DNA**

Genomic DNA was extracted using Qiagen (Hilden, Germany) or Promega (Madison, WI, USA) kits and quantified using the Qubit® system (life technologies, Carlsbad, USA).

**Capture bisulfite sequencing and DNA methylation mapping**

SeqCap Epi Enrichment System (Roche-NimbleGen) performed at the Institut de recherches cliniques de Montréal was used for targeted bisulfite sequencing of promoters and enhancers. Mouse target probes (mm9) were custom designed based on H3K4me1 and H3K4me3 signals from mouse public ChIP-seq data. A total of 83276 regions were targeted by the probes containing 64760111 base pairs. Biotinylated target probes were designed for both strands of bisulfite converted genomic DNA. Bisulfite-treated genomic DNA was ligated to methylated next-generation-sequencing-adaptors, hybridized to the biotinylated oligonucleotide probes followed by a series of washes of off-target DNA sequences and unbound DNA. Isolated DNA was amplified and sequenced (Illumina Hiseq 2000) with pair-end 50bp reads at Genome-Quebec Montreal, Canada.

**Analysis of differentially methylated cytosines**

FastQC assessed sequencing scores and other quality metrics. Trimmomatic was used to remove low scoring sequencing results and/or adapter contents. Sequences were aligned to the mm9 mouse reference genome using Bsmap v2.89 (6). Output data were strand-sorted, filtered, and deduplicated with Picard tools. Methylation levels and coverage levels were extracted with methratio.py command in Bsmap. Differential methylation cytosines were analyzed with methylKit R package (7) with FDR threshold of 0.2. Differentially methylated positions were annotated with HOMER (8).

**Gene-set analysis**

For gene-set analyses, selected genes were overlaid on the global molecular network developed from information contained in the Ingenuity Pathway knowledge base ([www.ingenuity.com](http://www.ingenuity.com), Qiagen).

**Pyrosequencing**

DNA samples were bisulfate converted (EZ DNA Methylation Gold Kit, Zymo Research, CA). The DNA fragment of interest was amplified by Touchdown PCR (TD-PCR) that involves two separate phases. For bisulfate DNA amplification we used *TaKaRa Taq DNA polymerase enzyme* (TaKaRa-Bio, USA). Pyrosequencing was performed using a PyroMark-Q24-Advanced system (Qiagen; primer information: see table S18) in accordance with the manufacturer's protocol. Methylated and unmethylated EpiTect control DNA samples (Qiagen) were used as controls for bisulfite conversion, amplification, and pyrosequencing. Data analysis was performed as described before (9) (Table S16).

**ChIP-bisulfite-Sequencing delineation of DNA methylation on GR binding regions in adult mice frontal cortex**
*Nr3c1^+/-^* and wild type mice were sacrificed, the brains were rapidly removed, frontal cortices were isolated, flesh frozen and stored at -80°C for later analysis.

Frontal cortices from eight male mice per group (one immunoprecipitation reaction/sample) were used for Glucocorticoid receptor Chromatin-immunoprecipitation followed by bisulfite-conversion and next-generation sequencing.

Samples were homogenized in 1 X PBS including 1% formaldehyde, and the homogenates were kept for 10 min at 25°C. Cross-linking reactions were stopped by the addition of glycine (125 mM) for 10 min at 25°C. Fixed chromatin samples were then homogenized in cell lysis solution (all solutions from ChIP-IT High-Sensitivity kit, Active-Motif and used according to manufacturer protocol) and centrifuge for 5 min at 3000 rpm, 4°C. Pellets were resuspended in RIPA-light solution (NaCl 150 mM, SDS 0.3%, Tris-HCl 50 mM (pH 8)) and sonicated using a covaris E220. Sonicated chromatin samples were then centrifuged for 15 min at 14000 rpm, 4°C. Pellets were resuspended in 1 ml of RIPA-light solution. Chromatin samples were pre-cleared with 50 µl of dynabeads protein G (Life Technologies) pre-blocked with BSA and incubated overnight at 4°C with an anti- GR antibody (1/100μg; Santa Cruz Biotechnologies; (H-300): sc-8992). Antibodies and chromatin were then mixed with 100 µl of dynabeads protein G for 3 hours at 4°C. The beads were then washed with RIPA-light solution and washing-solution. Protein–DNA complexes were eluted from the beads, de-cross-linked, treated with proteinase K and purified. The DNA concentration was determined by fluorometry on the Qubit system (Invitrogen). Equal amounts of DNA samples from all 8 mice per group were pooled and mixed.

Immunoprecipitated-DNA (200 ng) was used for DNA bisulfite-conversion using EZ DNA Methylation-GoldTM Kit (Zymo Research) according to the manufacturer recommended protocol.

Bisulfite-converted DNA from each experimental group (20 ng in 20 microliter) were used to prepare libraries and were sequenced on HiSeq 2000, 50 base-pairs pair-end at the IRCM (Institut de recherches cliniques de Montréal).

**Bioinformatic analysis of ChIP-bisulfite-sequencing data for GR regions**

Read quality assessed using Fastqc (http://www.bioinformatics.babraham.ac.uk/projects/fastqc/) confirmed high read quality and inconsequential levels of adapter contamination. Sequences were aligned to the mm9 mouse reference genome using Bsmap v2.89 (6). Output data were strand-sorted, filtered, and deduplicated with Picard tools. Next, methylation levels and coverage levels were extracted with methratio.py command in Bsmap. Differential methylation cytosines were analyzed with methylKit R package (7) with FDR threshold of 0.05. Differentially methylated positions were annotated with HOMER (8). The resulted list of adult frontal cortex GR-bound, differentially-methylated genes was intersected with the list of placental differentially-methylated genes. Significance of gene-lists overlap was determined with hyper-geometric test.

**References**

1. Tronche F, Kellendonk C, Reichardt HM, Schutz G. Genetic dissection of glucocorticoid receptor function in mice. Current opinion in genetics & development. 1998;8(5):532-8.

2. Tronche F, Kellendonk C, Kretz O, Gass P, Anlag K, Orban PC, et al. Disruption of the glucocorticoid receptor gene in the nervous system results in reduced anxiety. Nature genetics. 1999;23(1):99-103.

3. Ruder AM, Schmidt M, Ludiro A, Riva MA, Gass P. A technique for administering xenon gas anesthesia during surgical procedures in mice. Lab animal. 2014;43(11):405-9.

4. Chapman RH, Stern JM. Failure of severe maternal stress or ACTH during pregnancy to affect emotionality of male rat offspring: implications of litter effects for prenatal studies. Developmental psychobiology. 1979;12(3):255-67.

5. Pepin MC, Pothier F, Barden N. Impaired type II glucocorticoid-receptor function in mice bearing antisense RNA transgene. Nature. 1992;355(6362):725-8.

6. Xi Y, Li W. BSMAP: whole genome bisulfite sequence MAPping program. BMC bioinformatics. 2009;10:232.

7. Akalin A, Kormaksson M, Li S, Garrett-Bakelman FE, Figueroa ME, Melnick A, et al. methylKit: a comprehensive R package for the analysis of genome-wide DNA methylation profiles. Genome biology. 2012;13(10):R87.

8. Heinz S, Benner C, Spann N, Bertolino E, Lin YC, Laslo P, et al. Simple combinations of lineage-determining transcription factors prime cis-regulatory elements required for macrophage and B cell identities. Molecular cell. 2010;38(4):576-89.

9. Provencal N, Suderman MJ, Guillemin C, Massart R, Ruggiero A, Wang D, et al. The signature of maternal rearing in the methylome in rhesus macaque prefrontal cortex and T cells. The Journal of neuroscience : the official journal of the Society for Neuroscience. 2012;32(44):15626-42.
