# Supplementary material for: Fetal glucocorticoid receptor (Nr3c1) deficiency alters the landscape of DNA methylation of murine placenta in a sex-dependent manner and is associated to anxiety-like behavior in adulthood
Source: Transl Psychiatry. 2019 Jan 17;9:23. doi: 10.1038/s41398-018-0348-7 (PMC6336883; doi:10.1038/s41398-018-0348-7)
Supplement: Supplementary file 2 — Supplemental Figures [file 41398_2018_348_MOESM2_ESM.pptx]

## Slide 1
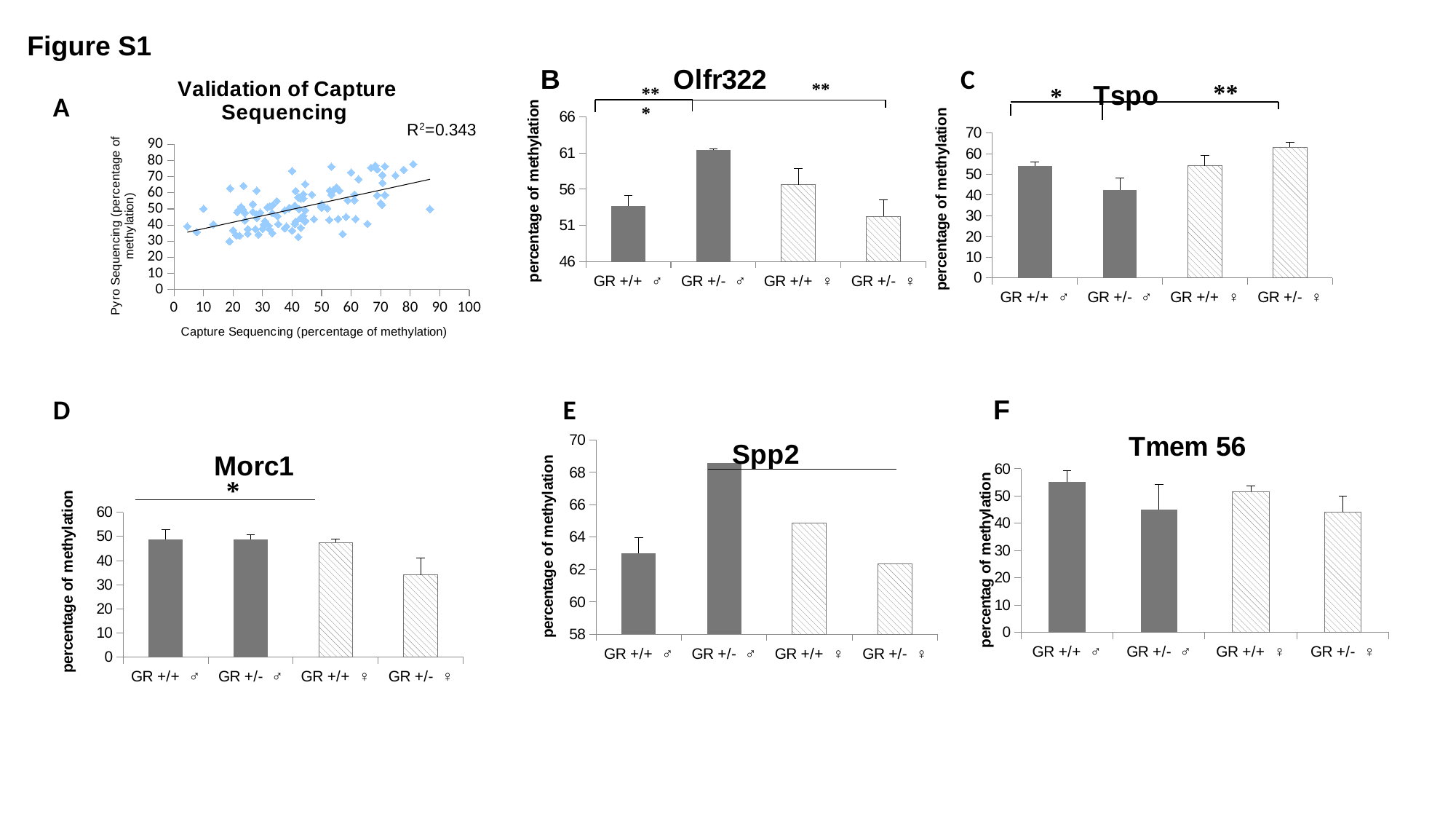

Figure S1
### Chart: Olfr322
| Category | |
|---|---|
| GR +/+ ♂ | 53.6367 |
| GR +/- ♂ | 61.38 |
| GR +/+ ♀ | 56.6015 |
| GR +/- ♀ | 52.2578 |B
C
### Chart: Validation of Capture Sequencing
| Category | Pyro Sequencing |
|---|---|
### Chart: Tspo
| Category | |
|---|---|
| GR +/+ ♂ | 54.0027 |
| GR +/- ♂ | 42.3489 |
| GR +/+ ♀ | 54.1262 |
| GR +/- ♀ | 63.0667 |A
D
E
F
### Chart: Tmem 56
| Category | |
|---|---|
| GR +/+ ♂ | 55.0673 |
| GR +/- ♂ | 45.1289 |
| GR +/+ ♀ | 51.6385 |
| GR +/- ♀ | 44.1833 |
### Chart: Spp2
| Category | |
|---|---|
| GR +/+ ♂ | 62.9933 |
| GR +/- ♂ | 68.5578 |
| GR +/+ ♀ | 64.8746 |
| GR +/- ♀ | 62.3556 |
### Chart: Morc1
| Category | |
|---|---|
| GR +/+ ♂ | 48.9127 |
| GR +/- ♂ | 48.6433 |
| GR +/+ ♀ | 47.52 |
| GR +/- ♀ | 34.1122 |

## Slide 2
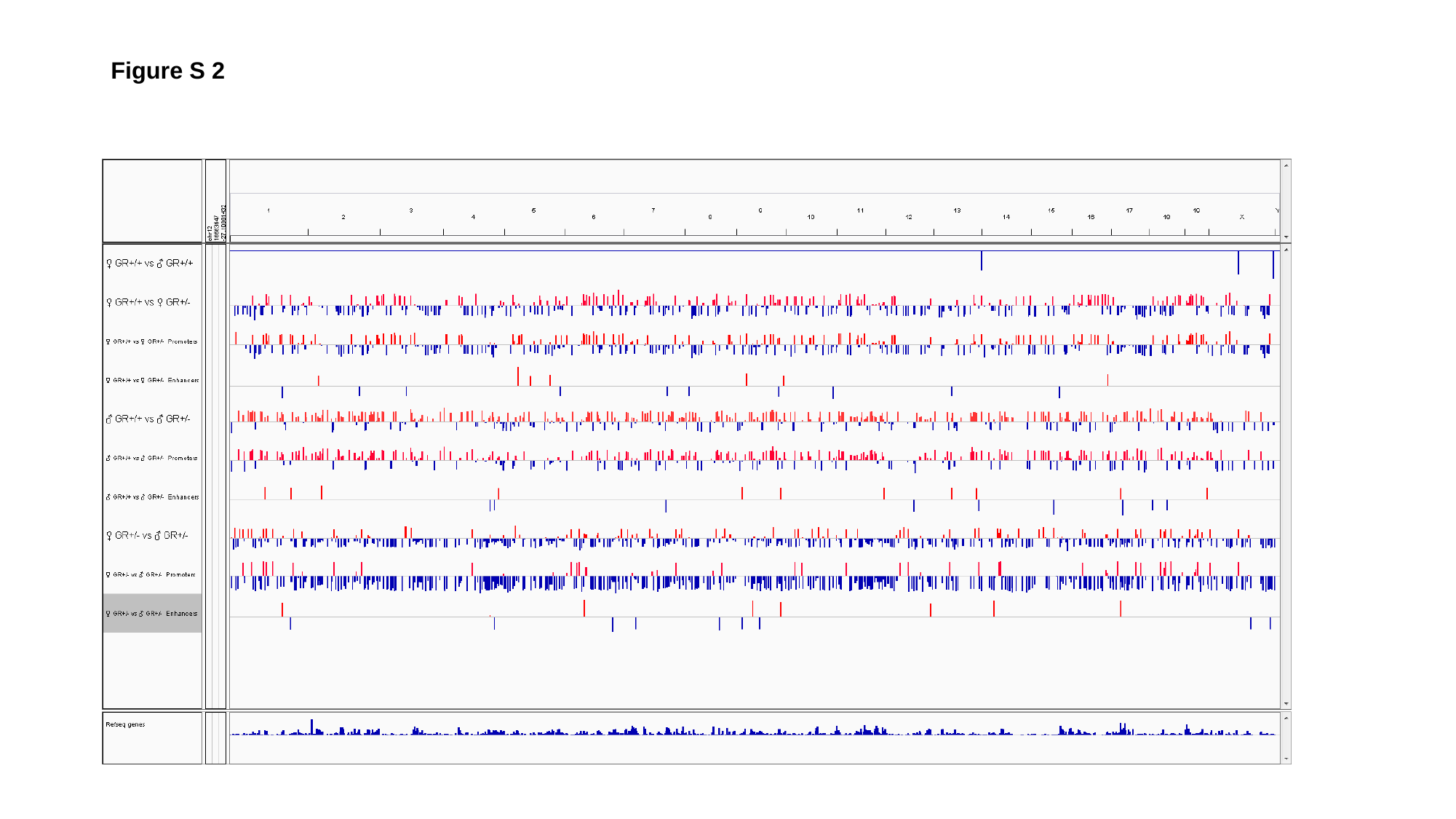

# Figure S 2

## Slide 3
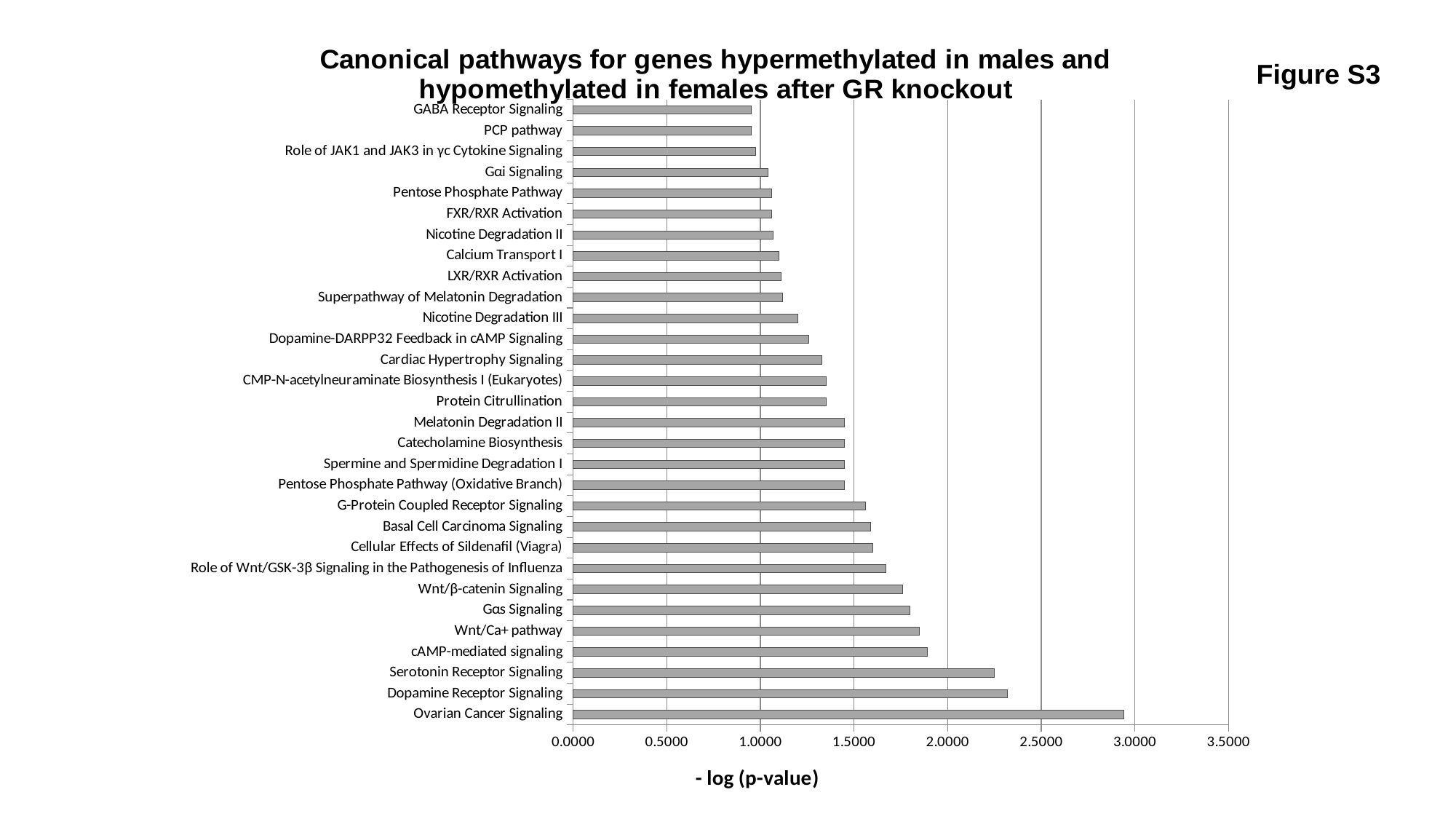

### Chart: Canonical pathways for genes hypermethylated in males and hypomethylated in females after GR knockout
| Category | |
|---|---|
| Ovarian Cancer Signaling | 2.94 |
| Dopamine Receptor Signaling | 2.32 |
| Serotonin Receptor Signaling | 2.25 |
| cAMP-mediated signaling | 1.89 |
| Wnt/Ca+ pathway | 1.85 |
| Gαs Signaling | 1.8 |
| Wnt/β-catenin Signaling | 1.76 |
| Role of Wnt/GSK-3β Signaling in the Pathogenesis of Influenza | 1.67 |
| Cellular Effects of Sildenafil (Viagra) | 1.6 |
| Basal Cell Carcinoma Signaling | 1.59 |
| G-Protein Coupled Receptor Signaling | 1.56 |
| Pentose Phosphate Pathway (Oxidative Branch) | 1.45 |
| Spermine and Spermidine Degradation I | 1.45 |
| Catecholamine Biosynthesis | 1.45 |
| Melatonin Degradation II | 1.45 |
| Protein Citrullination | 1.35 |
| CMP-N-acetylneuraminate Biosynthesis I (Eukaryotes) | 1.35 |
| Cardiac Hypertrophy Signaling | 1.33 |
| Dopamine-DARPP32 Feedback in cAMP Signaling | 1.26 |
| Nicotine Degradation III | 1.2 |
| Superpathway of Melatonin Degradation | 1.12 |
| LXR/RXR Activation | 1.11 |
| Calcium Transport I | 1.1 |
| Nicotine Degradation II | 1.07 |
| FXR/RXR Activation | 1.06 |
| Pentose Phosphate Pathway | 1.06 |
| Gαi Signaling | 1.04 |
| Role of JAK1 and JAK3 in γc Cytokine Signaling | 0.976 |
| PCP pathway | 0.953 |
| GABA Receptor Signaling | 0.953 |Figure S3

## Slide 4
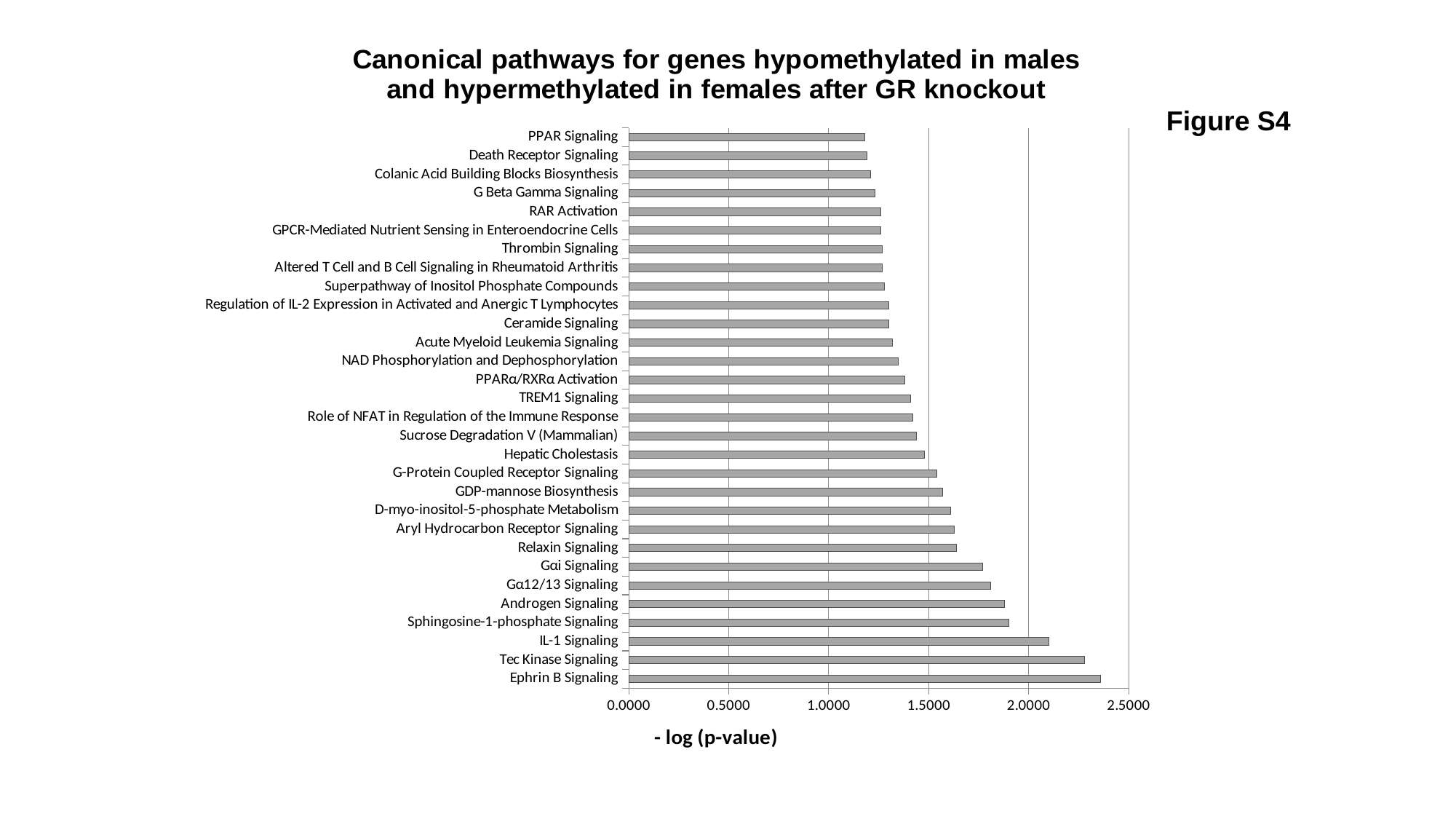

### Chart: Canonical pathways for genes hypomethylated in males and hypermethylated in females after GR knockout
| Category | |
|---|---|
| Ephrin B Signaling | 2.36 |
| Tec Kinase Signaling | 2.28 |
| IL-1 Signaling | 2.1 |
| Sphingosine-1-phosphate Signaling | 1.9 |
| Androgen Signaling | 1.88 |
| Gα12/13 Signaling | 1.81 |
| Gαi Signaling | 1.77 |
| Relaxin Signaling | 1.64 |
| Aryl Hydrocarbon Receptor Signaling | 1.63 |
| D-myo-inositol-5-phosphate Metabolism | 1.61 |
| GDP-mannose Biosynthesis | 1.57 |
| G-Protein Coupled Receptor Signaling | 1.54 |
| Hepatic Cholestasis | 1.48 |
| Sucrose Degradation V (Mammalian) | 1.44 |
| Role of NFAT in Regulation of the Immune Response | 1.42 |
| TREM1 Signaling | 1.41 |
| PPARα/RXRα Activation | 1.38 |
| NAD Phosphorylation and Dephosphorylation | 1.35 |
| Acute Myeloid Leukemia Signaling | 1.32 |
| Ceramide Signaling | 1.3 |
| Regulation of IL-2 Expression in Activated and Anergic T Lymphocytes | 1.3 |
| Superpathway of Inositol Phosphate Compounds | 1.28 |
| Altered T Cell and B Cell Signaling in Rheumatoid Arthritis | 1.27 |
| Thrombin Signaling | 1.27 |
| GPCR-Mediated Nutrient Sensing in Enteroendocrine Cells | 1.26 |
| RAR Activation | 1.26 |
| G Beta Gamma Signaling | 1.23 |
| Colanic Acid Building Blocks Biosynthesis | 1.21 |
| Death Receptor Signaling | 1.19 |
| PPAR Signaling | 1.18 |Figure S4
