# Supplementary material for: Fetal glucocorticoid receptor (Nr3c1) deficiency alters the landscape of DNA methylation of murine placenta in a sex-dependent manner and is associated to anxiety-like behavior in adulthood
Source: Transl Psychiatry. 2019 Jan 17;9:23. doi: 10.1038/s41398-018-0348-7 (PMC6336883; doi:10.1038/s41398-018-0348-7)
Supplement: Supplementary file 3 — Supplemental Tables [file 41398_2018_348_MOESM3_ESM.docx]

Supplemental Tables

**Table S1: Animals sex and genotype per group**

Table S2: Maternal Care Behavior

**TableS3: Ethogram**

| **Mouse Nr.:** | **PND:** | | | | | | | | | |  |  |  |  |  |  |  |  |  |  |
| --- | --- | --- | --- | --- | --- | --- | --- | --- | --- | --- | --- | --- | --- | --- | --- | --- | --- | --- | --- | --- |
| **Time** |  | | | | | | | | | |  | | | | | | | | | |
|  | 1 | 2 | 3 | 4 | 5 | 6 | 7 | 8 | 9 | 10 | 1 | 2 | 3 | 4 | 5 | 6 | 7 | 8 | 9 | 10 |
| **in nest** |  |  |  |  |  |  |  |  |  |  |  |  |  |  |  |  |  |  |  |  |
| licking / grooming |  |  |  |  |  |  |  |  |  |  |  |  |  |  |  |  |  |  |  |  |
| active / passive nursing |  |  |  |  |  |  |  |  |  |  |  |  |  |  |  |  |  |  |  |  |
| nest building |  |  |  |  |  |  |  |  |  |  |  |  |  |  |  |  |  |  |  |  |
| eating / drinking |  |  |  |  |  |  |  |  |  |  |  |  |  |  |  |  |  |  |  |  |
| self-grooming |  |  |  |  |  |  |  |  |  |  |  |  |  |  |  |  |  |  |  |  |
| exploring |  |  |  |  |  |  |  |  |  |  |  |  |  |  |  |  |  |  |  |  |
| **out of nest** |  |  |  |  |  |  |  |  |  |  |  |  |  |  |  |  |  |  |  |  |
| eating / drinking |  |  |  |  |  |  |  |  |  |  |  |  |  |  |  |  |  |  |  |  |
| self-grooming |  |  |  |  |  |  |  |  |  |  |  |  |  |  |  |  |  |  |  |  |
| sleeping /resting |  |  |  |  |  |  |  |  |  |  |  |  |  |  |  |  |  |  |  |  |
| climbing / digging / exploring |  |  |  |  |  |  |  |  |  |  |  |  |  |  |  |  |  |  |  |  |
| Stereotypic behaviors |  |  |  |  |  |  |  |  |  |  |  |  |  |  |  |  |  |  |  |  |
| Maternal moving |  |  |  |  |  |  |  |  |  |  |  |  |  |  |  |  |  |  |  |  |
| pups out of nest |  |  |  |  |  |  |  |  |  |  |  |  |  |  |  |  |  |  |  |  |
| Other |  |  |  |  |  |  |  |  |  |  |  |  |  |  |  |  |  |  |  |  |
| undetectable (poor visibility) |  |  |  |  |  |  |  |  |  |  |  |  |  |  |  |  |  |  |  |  |

**Table S4: Number of CpG, CHG and CHH differentially methylated in GR+/- fetal placenta (Number of sites for 15 reads in the GR groups (q=0.2) for different delta beta values; in bold: values chosen for this study)**

| **Site of methylation** | **Comparison** | **Delta beta 30 %** | **Delta beta 25 %** | **Delta beta 20 %** | **Delta beta 15 %** | **Delta beta 10 %** | **Delta beta 5 %** |
| --- | --- | --- | --- | --- | --- | --- | --- |
| **CpG** | ♀ GR+/+ vs. ♂ GR+/+ | 2 | 3 | **6** | 6 | 6 | 6 |
|  | ♀ GR+/+ vs. GR +/- | 42 | 287 | **1,754** | 8,423 | 33,240 | 109,368 |
|  | ♂ GR+/+ vs. GR +/- | 42 | 311 | **1,829** | 9,109 | 34,662 | 112,361 |
|  | ♀ GR+/- vs. ♂ GR+/- | 73 | 471 | **2,433** | 10,766 | 38,006 | 113,561 |
| **CHG** | ♀ GR+/+ vs. ♂ GR+/+ | 0 | 0 | **0** | 1 | 24 | 967 |
|  | ♀ GR+/+ vs. GR +/- | 0 | 0 | **0** | 1 | 30 | 984 |
|  | ♂ GR+/+ vs. GR +/- | 0 | 0 | **0** | 5 | 17 | 1,032 |
|  | ♀ GR+/- vs. ♂ GR+/- | 0 | 0 | **0** | 0 | 18 | 1,035 |
| **CHH** | ♀ GR+/+ vs. ♂ GR+/+ | 0 | 1 | **4** | 15 | 207 | 4,312 |
|  | ♀ GR+/+ vs. GR +/- | 1 | 1 | **2** | 17 | 223 | 4,475 |
|  | ♂ GR+/+ vs. GR +/- | 0 | 0 | **1** | 21 | 234 | 4,413 |
|  | ♀ GR+/- vs. ♂ GR+/- | 0 | 1 | **3** | 27 | 249 | 4,494 |

**Table S5: list of genes hypomethylated in both sexes after GR knockout**

| Gene name | Chr. | Pos. ♂ | Delta beta ♂ | Distance to TSS ♂ | Pos. ♀ | Delta beta ♀ | Distance to TSS ♀ |
| --- | --- | --- | --- | --- | --- | --- | --- |
| Abhd6 | chr14 | 8833738 | -21,58651189 | -1678 | 8833658 | -31,37254902 | -1758 |
| Ahsg | chr16 | 22891401 | -23,67941712 | -687 | 22895714 | -27,20533729 | 3626 |
| Ahsg | chr16 | 22895577 | 21,89957653 | 3489 | - | - | - |
| Asphd2 | chr5 | 112821621 | -21,4951715 | -388 | 112821526 | -24,60784314 | -293 |
| Asphd2 | chr5 | 112820615 | 20,09397457 | 618 |  |  |  |
| AU015791 | chr12 | 106750433 | -20,34482759 | -1210 | 106750767 | -20,97601642 | -876 |
| AU015791 | chr12 | - | - | - | 106750424 | 20,68965517 | -1219 |
| B930025P03Rik | chr8 | 10883157 | -27,62908325 | -703 | 10644911 | -21,58528852 | 237543 |
| B930025P03Rik | chr8 | - | - | - | 10883249 | 20,08547009 | -795 |
| B930025P03Rik | chr8 | - | - | - | 10646651 | 26,9858156 | 235803 |
| C77080 | chr4 | 128899433 | -25,6980057 | 5722 | 128901138 | -21,9047619 | 4017 |
| C77080 | chr4 | 128917906 | 29,03225806 | -1444 | 128901150 | -21,84628237 | 4005 |
| C77080 | chr4 | - | - | - | 128903171 | 20,11481056 | 1984 |
| Cadm4 | chr7 | 25265529 | -20,49441786 | -1513 | 25265586 | -23,40301974 | -1456 |
| Cdr1 | chrX | 58437471 | -25,02311248 | 1262 | 58438022 | -21,39415839 | 711 |
| Dst | chr1 | 34245672 | -23,20884146 | 28497 | 34234868 | -22,02531646 | 17693 |
| Dst | chr1 | 34246343 | 20,02262443 | 29168 | 34234895 | -21,68560606 | 17720 |
| Dst | chr1 | 34249160 | 21,875 | 31985 | 34246351 | -20,39191672 | 29176 |
| Dst | chr1 | 34248311 | 24,29233145 | 31136 | 34234889 | -20,25974026 | 17714 |
| Eif5a2 | chr3 | 28679670 | -20,37221668 | -563 | 28646564 | -22,23989096 | -33669 |
| Fetub | chr16 | 22920361 | -20,86286595 | 66 | 22917993 | -20,09760426 | -462 |
| Fshr | chr17 | 89601266 | -21,92028986 | -1251 | 89601266 | -21,12280702 | -1251 |
| Fzd1 | chr5 | 4665724 | -22,36098721 | 92492 | 4666221 | -23,01587302 | 91995 |
| Galnt15 | chr14 | 32859682 | -22,82608696 | 17393 | 32869548 | -20,69230769 | 27259 |
| Galnt15 | chr14 | - | - | - | 32842051 | -20,55660974 | -238 |
| Gm13283 | chr4 | 88405501 | -20,42954426 | -1177 | 88405502 | -20,08978676 | -1176 |
| Grm4 | chr17 | 27641535 | -20,27777778 | -1286 | 27641487 | -25,87570621 | -1238 |
| Gsx2 | chr5 | 75471149 | -21,72792855 | -477 | 75473178 | -23,24561404 | 1552 |
| Gsx2 | chr5 | 75472999 | 20,2764977 | 1373 | - | - |  |
| Helz2 | chr2 | 180969046 | -21,30378096 | 7686 | 180969980 | -20,13680347 | 6752 |
| Helz2 | chr2 | 180969472 | 25,3655914 | 7260 | 180968883 | 22,27272727 | 7849 |
| Hoxd13 | chr2 | 74505840 | -22,29262673 | -527 | 74505515 | -21,34894091 | -852 |
| Jak3 | chr8 | 74204645 | -21,77871148 | 4363 | 74204602 | -22,87735849 | 4320 |
| Kcna5 | chr6 | 126483628 | -21,41176471 | 1945 | 126484362 | -21,45054233 | 1211 |
| Klhl38 | chr15 | 58157022 | -28,13519814 | -1298 | 58154255 | -27,45098039 | 1469 |
| Lbp | chr2 | 158150304 | -23,28767123 | 18075 | 158150410 | -20,46398046 | 18181 |
| Ly86 | chr13 | 37437254 | -22,85815855 | 40 | 37436388 | -22,22222222 | -826 |
| Map3k14 | chr11 | 103129882 | -24,09502262 | -1167 | 103129883 | -23,16017316 | -1168 |
| Mfrp | chr9 | 43911039 | -20,58936826 | 1186 | 43909735 | -24,07270941 | -118 |
| Mir1906-1 | chr12 | 110782249 | -29,25390179 | -502 | 110781986 | -25,33170035 | -765 |
| Mir1906-1 | chr12 | - | - | - | 110782598 | -24,16666667 | -153 |
| Mir6387 | chr12 | 15808107 | -20,88235294 | -200 | 15812609 | -20,00755715 | -4702 |
| Mir7678 | chr2 | 164180983 | -20,13808976 | -864 | 164180530 | -24,14383562 | -411 |
| Mir7678 | chr2 | - | - | - | 164180541 | -20,28735632 | -422 |
| Mst1r | chr9 | 107810403 | -20,04301075 | 1183 | 107809538 | -20,3320802 | 318 |
| Mtus1 | chr8 | 42141531 | -24,28571429 | -1381 | 42220229 | -20,43881857 | -1149 |
| Nr3c1 | chr18 | 39588381 | -24,69135802 | 58518 | 39646957 | -21,00015825 | -58 |
| Papln | chr12 | 85103234 | -29,75423046 | -1350 | 85104291 | -22,2826087 | -293 |
| Paqr4 | chr17 | 23878144 | -22,49382716 | -847 | 23878142 | -23,38516746 | -845 |
| Paqr4 | chr17 | - | - | - | 23878144 | -22,42524917 | -847 |
| Ppm1f | chr16 | 16932084 | -26,04761905 | 35522 | 16903496 | -24,09007594 | 6934 |
| Qrich2 | chr11 | 116316848 | -24,05529954 | -1187 | 116316497 | -20,60012438 | -836 |
| Rbpms2 | chr9 | 65489632 | -22,37288136 | 11243 | 65489317 | -20,2247191 | 10928 |
| Rtn1 | chr12 | 73464721 | -22,25534029 | 45320 | 73336974 | -22,17653509 | -241 |
| Rtn1 | chr12 | 73405365 | 21,72670097 | -67651 | 73404971 | -20,14589144 | -67257 |
| Rusc1 | chr3 | 88891797 | -25,92592593 | 2156 | 88891797 | -30,85255066 | 2156 |
| Rusc1 | chr3 | - | - | - | 88891798 | 21.2121212121212-2 | 2155 |
| Schip1 | chr3 | 68376492 | -24,88207547 | 202 | 68027991 | -21,69059011 | 159267 |
| Schip1 | chr3 | 68228924 | -21,38621201 | -43181 | 68296585 | -20,44807966 | -1518 |
| Schip1 | chr3 | 67868740 | 20,04201681 | 16 | 67868401 | 24,75106686 | -323 |
| Schip1 | chr3 | 68271612 | 23,52112676 | -493 |  |  |  |
| Sorbs3 | chr14 | 70593389 | -20,625 | -1336 | 70608473 | -21,84210526 | -995 |
| Tbc1d1 | chr5 | 64564925 | -25,69230769 | 13475 | 64654792 | -27,32497388 | 103342 |
| Tcam1 | chr11 | 106147790 | -21,67046565 | 9804 | 106136440 | -23,83126767 | -1546 |
| Tcam1 | chr11 | - | - | - | 106137581 | 27,57885763 | -405 |
| Tgm6 | chr2 | 129937710 | -22,33589088 | -11311 | 129948883 | -24,95777027 | -138 |
| Tiam2 | chr17 | 3483231 | -28,33333333 | -259 | 3414182 | -24,71794872 | 16975 |
| Tiam2 | chr17 | 3397109 | -20,75949367 | -98 | 3483336 | 20,95238095 | -154 |
| Tiam2 | chr17 | - | - | - | 3483205 | 22,23707665 | -285 |
| Tm4sf4 | chr3 | 57286074 | -29,13543228 | 56742 | 57229495 | -22,51521298 | 163 |
| Tm7sf2 | chr19 | 6068370 | -20,04504505 | -520 | 6070743 | -21,18702553 | -2893 |
| Trim69 | chr2 | 121984673 | -21,96620584 | -1763 | 121985769 | -25,85895118 | -667 |
| Trim69 | chr2 | - | - | - | 121985765 | -20,71800493 | -671 |
| Tsc22d1 | chr14 | 76887484 | -21,84035477 | -759 | 76903487 | -26,06060606 | -830 |
| Tsc22d1 | chr14 | 76887813 | 20,71298465 | -430 |  |  |  |
| Tspo | chr15 | 83393244 | -26,67296489 | -759 | 83392838 | -20,33333333 | -1165 |
| 5033406O09Rik | chr12 | 113184383 | -23,39784946 | -1690 | 113184383 | -20,23772769 | -1690 |

**Table S6: list of genes hypermethylated in both sexes after GR knockout**

| Gene name | Chr. | Pos. ♀ | Delta beta ♀ | Distance to TSS ♀ | Pos. ♂ | Delta beta ♂ | Distance to TSS ♂ |
| --- | --- | --- | --- | --- | --- | --- | --- |
| Ankrd2 | chr19 | 42110266 | 22,26631393 | -262 | 42108420 | 20,50189394 | -2108 |
| Ankrd2 | chr19 | 42110253 | 25,22702104 | -275 | - | - | - |
| Ankrd46 | chr15 | 36415721 | 23,44771242 | 10825 | 36408185 | 21,95444915 | 18361 |
| App | chr16 | 85103418 | 25 | 70534 | 85097100 | 22,08955224 | 76852 |
| Aurka | chr2 | 172195122 | 23,283859 | 884 | 172194588 | 21,6194332 | 1418 |
| B2m | chr2 | 121972903 | 21,9924812 | -520 | 121972336 | 25,17361111 | -1087 |
| Bpi | chr2 | 158083811 | 24,1948154 | -166 | 158083136 | 27,14285714 | -841 |
| C77080 | chr4 | 128901138 | -21,9047619 | 4017 | 128899433 | -25,6980057 | 5722 |
| C77080 | chr4 | 128901150 | -21,84628237 | 4005 | 128917906 | 29,03225806 | -1444 |
| C77080 | chr4 | 128903171 | 20,11481056 | 1984 | - | - | - |
| Cacnb1 | chr11 | 97880999 | -23,96377569 | -1313 | 97880999 | 30,06333775 | -1313 |
| Cacnb1 | chr11 | 97881002 | 20,02956393 | -1316 | - | - | - |
| Casc1 | chr6 | 145122995 | 20,46098621 | 36495 | 145123053 | 20,90126812 | 36437 |
| Ccm2l | chr2 | 152890519 | 22,24561404 | -1172 | 152889716 | 21,2622549 | -1975 |
| Ccser1 | chr6 | 61128579 | 20,3346856 | -1740 | 61129103 | 20,46736503 | -1216 |
| Cenpb | chr2 | 131004680 | 23,72512438 | 1068 | 131007900 | 20,22147328 | -2152 |
| Chst5 | chr8 | 114435797 | 21.875-4 | -1698 | 114434529 | 20,39800995 | -430 |
| Col6a4 | chr9 | 105977189 | 22,50443477 | 21833 | 105999426 | 25,89962121 | -404 |
| Crybb3 | chr5 | 113510728 | 23,31932773 | -124 | 113510661 | 20.8333333333333-4 | -57 |
| Dgke | chr11 | 88921054 | 28,18627451 | 1008 | 88922836 | 20,89552239 | -774 |
| Dgke | chr11 | - | - | - | 88922861 | 22,0821662 | -799 |
| Dnah2 | chr11 | 69334245 | 20,75471698 | 28365 | 69363589 | 22,0959596 | -979 |
| Edn2 | chr4 | 119833956 | 24,42645074 | -73 | 119833378 | 27,82658518 | -651 |
| Emp3 | chr7 | 53173444 | 20,66666667 | 2775 | 53171511 | 20.7589285714286-2 | 4708 |
| Fam105b | chr15 | 27558258 | 21,16244411 | 2190 | 27558317 | 22,71794872 | 2131 |
| Fam64a | chr11 | 71856665 | 32,15940686 | 661 | 71858641 | 21,98748044 | 2637 |
| Fam83h | chr15 | 75833084 | -29,05982906 | 6844 | 75834751 | 21,88697318 | 5177 |
| Fam83h | chr15 | 75834743 | 21,53846154 | 5185 | - | - | - |
| Fam83h | chr15 | 75834753 | 26,10294118 | 5175 | - | - | - |
| Fes | chr7 | 87535803 | 20,32183908 | -2971 | 87535928 | 20,57142857 | -3096 |
| Fes | chr7 |  |  |  | 87535919 | 23,38914257 | -3087 |
| Flt1 | chr5 | 148539285 | 20,33467202 | -1721 | 148583517 | 21,37681159 | -45953 |
| Frem1 | chr4 | 82618191 | 26,16438356 | 48815 | 82618171 | 25,4978355 | 48835 |
| Frem1 | chr4 | - | - | - | 82668346 | -31,18908382 | -1340 |
| Gm7120 | chr13 | 120276435 | 24,30875576 | -411 | 120276493 | 23,54048964 | -353 |
| Gpr39 | chr1 | 127574441 | 22,4137931 | 868 | 127574263 | -20,08097166 | 690 |
| Gpr39 | chr1 | - | - | - | 127573958 | 20,10869565 | 385 |
| Gpr39 | chr1 | - | - | - | 127573858 | 24,70034247 | 285 |
| Gsn | chr2 | 35121592 | 23,03232999 | -672 | 35121947 | 22,21468927 | -317 |
| Helz2 | chr2 | 180969980 | -20,13680347 | 6752 | 180969046 | -21,30378096 | 7686 |
| Helz2 | chr2 | 180968883 | 22,27272727 | 7849 | 180969472 | 25,3655914 | 7260 |
| Icam4 | chr9 | 20834530 | 21,61089053 | 713 | 20832064 | 22,29654404 | -1753 |
| Igsf8 | chr1 | 174246332 | 24,21434327 | 3794 | 174248431 | 26,9005848 | 5893 |
| Impa1 | chr3 | 10332075 | 24,51676046 | -636 | 10328422 | 20,27359781 | 3017 |
| Khk | chr5 | 31223211 | 24,35443038 | -1057 | 31222519 | -22,90960452 | -1749 |
| Khk | chr5 | - | - | - | 31222887 | -21,71412137 | -1381 |
| Khk | chr5 | - | - | - | 31223028 | 30,59701493 | -1240 |
| Krt80 | chr15 | 101200465 | 20,90524259 | 91 | 101200444 | -20,65831491 | 112 |
| Krt80 | chr15 | - | - | - | 101200467 | 23,71794872 | 89 |
| Lpin1 | chr12 | 16597859 | 20,63492063 | -1283 | 16597223 | 21,3240057 | -647 |
| Map3k12 | chr15 | 102333118 | 26,65092277 | 14317 | 102331803 | 20,67610063 | 15632 |
| Map3k12 | chr15 | - | - | - | 102331809 | 20,70660522 | 15626 |
| Marveld3 | chr8 | 112472069 | 24,16666667 | 14036 | 112487512 | 24,14679757 | -1407 |
| Mir148a | chr6 | 51165962 | 21,03386809 | 53947 | 51165853 | 20,40271443 | 54056 |
| Mir467a-4 | chr2 | 10419294 | 23,27210461 | -726 | 10397968 | 22,62996942 | -1 |
| Mir6366 | chr16 | 18165986 | -20,81818182 | -723 | 18166879 | 24,44444444 | -1616 |
| Mir6366 | chr16 | 18151344 | -20,47769582 | 13919 | - | - | - |
| Mir6366 | chr16 | 18151638 | 21,20220814 | 13625 | - | - | - |
| Mir7078 | chr8 | 119981505 | 20,49786629 | -1660 | 119981505 | 20,95712507 | -1660 |
| Mir8093 | chr2 | 32543636 | 20,13134058 | -350 | 32534612 | 23,03499713 | 8674 |
| Mir8093 | chr2 | 32544106 | 24,28432327 | -820 |  |  |  |
| Mir8103 | chr11 | 96924466 | 22,67759563 | -616 | 96924647 | 26,37362637 | -435 |
| Mixl1 | chr1 | 182625019 | 26,49136578 | 2146 | 182625018 | 22,10187354 | 2147 |
| Nav2 | chr7 | 56414373 | 20,44334975 | -87186 | 56659212 | 26,486213 | 157653 |
| Olfr1392 | chr11 | 49106361 | 22,14673913 | -438 | 49106492 | 21,12820513 | -307 |
| Pdgfrb | chr18 | 61225454 | 21,87639946 | 20650 | 61220895 | 22,69619907 | 16091 |
| Pgc | chr17 | 47862823 | 20,59852312 | -968 | 47863799 | 24,93481095 | 8 |
| Pgc | chr17 | - | - | - | 47863804 | 27,20736267 | 13 |
| Plec | chr15 | 76037629 | 21,4559387 | -878 | 76032093 | 20.2898550724638-2 | -1056 |
| Prkcdbp | chr7 | 112629589 | 21,07865169 | 1122 | 112631377 | 21,65730337 | -666 |
| Prpsap2 | chr11 | 61576004 | 20,06410256 | -414 | 61575976 | 21,00961538 | -386 |
| Prss8 | chr7 | 135077579 | 21.6133942161339-2 | -3952 | 135074408 | 28,71572872 | -781 |
| Ptgr2 | chr12 | 85625361 | 21,68162494 | -885 | 85625479 | 20,23283822 | -767 |
| Rn4.5s | chr6 | 47611209 | 22,87644788 | -1795 | 47624151 | -22,87669195 | -1753 |
| Rn4.5s | chr6 | 47687937 | 23,11066127 | 6293 | 47687452 | -22,34408602 | 6778 |
| Rn4.5s | chr6 | 47692180 | 23,90678188 | 2050 | 47718702 | -20,81280788 | -15778 |
| Rn4.5s | chr6 | 47603601 | 27,68764486 | 1492 | 47614938 | 20,67873303 | -1184 |
| Rn4.5s | chr6 | - | - | - | 47598749 | 22,25274725 | 6344 |
| Rn4.5s | chr6 | - | - | - | 47704338 | 22,97607342 | -1414 |
| Rn4.5s | chr6 | - | - | - | 47715156 | 24,31818182 | -12232 |
| Rn4.5s | chr6 | - | - | - | 47633865 | 22,222222 | 1510 |
| Sap25 | chr5 | 138081111 | 28,58823529 | -1590 | 138081347 | 20,64197531 | -1354 |
| Schip1 | chr3 | 68027991 | -21,69059011 | 159267 | 68376492 | -24,88207547 | 202 |
| Schip1 | chr3 | 68296585 | -20,44807966 | -1518 | 68228924 | -21,38621201 | -43181 |
| Schip1 | chr3 | 67868401 | 24,75106686 | -323 | 67868740 | 20,04201681 | 16 |
| Schip1 | chr3 | - | - | - | 68271612 | 23,52112676 | -493 |
| Sipa1l2 | chr8 | 127876331 | 20,75837743 | 140279 | 128016693 | 26,28689087 | -83 |
| Slco3a1 | chr7 | 81649304 | 20,3042328 | 50362 | 81649225 | 21.4285714285714-8 | 50441 |
| Slco3a1 | chr7 | 81636854 | 22,55892256 | 62812 | - | - | - |
| Snora36b | chr1 | 187066325 | 21,40790743 | -480 | 187066742 | 31,07890499 | -63 |
| Snora47 | chr13 | 96106109 | 21,66780588 | 5543 | 96106133 | 20,55555556 | 5567 |
| Snord85 | chr4 | 130305669 | 25 | 116 | 130305351 | 22,32854864 | -202 |
| Spaca4 | chr7 | 52983171 | -24,62546816 | -1985 | 52982979 | 21,69753086 | -1793 |
| Spaca4 | chr7 | 52982031 | 22.1433814533888-2 | -845 | - | - | - |
| St3gal3 | chr4 | 117808385 | 20,88618299 | -834 | 117825860 | 20.3174603174603-2 | -18309 |
| Sumo2 | chr11 | 115398391 | 21,81372549 | -847 | 115398391 | 23,78731343 | -847 |
| Tmem63a | chr1 | 182871101 | 24,58970006 | -1548 | 182871014 | 20,76923077 | -1635 |
| Tmem87b | chr2 | 128609662 | 24,24698795 | -34377 | 128609630 | 26,6190719 | -34409 |
| Tnks1bp1 | chr2 | 84902180 | -23,77819549 | 11563 | 84903488 | 22,68578879 | 12871 |
| Tnks1bp1 | chr2 | 84902831 | -21,4391912 | 12214 | - | - | - |
| Tnks1bp1 | chr2 | 84903085 | 22,54901961 | 12468 | - | - | - |
| Tns3 | chr11 | 8566007 | 28,64864865 | -1469 | 8393219 | 21,52077451 | 171319 |
| Wdfy3 | chr5 | 102372070 | 20,86397059 | 126870 | 102366537 | 22,37762238 | 132403 |
| 1700060C20Rik | chr2 | 158016935 | 23,36592898 | -809 | 158016713 | 21,01010101 | -1031 |
| 1700125H03Rik | chr8 | 70892402 | 32,58928571 | 44 | 70854988 | 20,87420043 | -37370 |
| 4930487D11Rik | chr5 | 38735225 | 21,29098361 | -403 | 38735225 | 21,63292254 | -403 |
| 4930487D11Rik | chr5 | - | - | - | 38742549 | 24,04371585 | 6921 |
| 9530052E02Rik | chr8 | 11006782 | 21,58848614 | -1068 | 11005631 | 20,70707071 | -2219 |
| 9530052E02Rik | chr8 | - | - | - | 11006139 | 21.4285714285714-10 | -1711 |

**Table S7: list of genes hypermethylated in males and hypomethylated in females after GR knockout**

| Gene name | Chr. | Pos. ♂ | Delta beta ♂ | Distance to TSS ♂ | Pos. ♀ | Delta beta ♀ | Distance to TSS ♀ |
| --- | --- | --- | --- | --- | --- | --- | --- |
| Acsf2 | chr11 | 94464298 | 20,08101852 | -1198 | 94464484 | -22,83333333 | -1384 |
| Ahsg | chr16 | 22891401 | -23,67941712 | -687 | 22895714 | -27,20533729 | 3626 |
| Ahsg | chr16 | 22895577 | 21,89957653 | 3489 | - | - | - |
| Anapc2 | chr2 | 25129618 | 20.8333333333333-3 | 1632 | 25129387 | -22,39482201 | 1401 |
| Ank1 | chr8 | 24206142 | 20,31312726 | 37411 | 24074019 | -24,84848485 | -11289 |
| Ank1 | chr8 | 24144319 | 21,39406487 | -1252 | - | - | - |
| Armcx4 | chrX | 131225765 | 21,12890923 | 4707 | 131225942 | -21,26188418 | 4884 |
| Arvcf | chr16 | 18391243 | 22,90730337 | -1413 | 18347829 | -20,18518519 | -446 |
| Asphd2 | chr5 | 112821621 | -21,4951715 | -388 | 112821526 | -24,60784314 | -293 |
| Asphd2 | chr5 | 112820615 | 20,09397457 | 618 | - | - | - |
| Atxn1 | chr13 | 46046009 | 21,51100376 | 14351 | 46046748 | -20,26455026 | 13612 |
| B230208H11Rik | chr10 | 12661174 | 22,14285714 | -18249 | 12644301 | -32,8631139 | -1376 |
| C77080 | chr4 | 128899433 | -25,6980057 | 5722 | 128901150 | -21,84628237 | 4005 |
| C77080 | chr4 | 128917906 | 29,03225806 | -1444 | 128903171 | 20,11481056 | 1984 |
| Cacna1d | chr14 | 31304601 | 20,85416667 | -259 | 31304843 | -22,69055146 | -501 |
| Cacnb1 | chr11 | 97880999 | 30,06333775 | -1313 | 97880999 | -23,96377569 | -1313 |
| Cacnb1 | chr11 | - | - | - | 97881002 | 20,02956393 | -1316 |
| Ccdc162 | chr10 | 41308312 | 21,92949907 | -753 | 41308281 | -22,191864 | -722 |
| Celsr2 | chr3 | 108210094 | 24,07407407 | 8318 | 108210260 | -33,5071708 | 8152 |
| Celsr2 | chr3 | 108209988 | 24,64768479 | 8424 | 108217774 | -27,81493868 | 638 |
| Celsr2 | chr3 | - | - | - | 108217467 | -22,01450443 | 945 |
| Ctxn3 | chr18 | 57627456 | 20,3030303 | -684 | 57588730 | -27,00854701 | -39410 |
| Cul7 | chr17 | 46786731 | 21,03632779 | -556 | 46785709 | -20,59259259 | -1578 |
| Cyp1a1 | chr9 | 57543785 | 20,1486698 | -1635 | 57544921 | -22,11864407 | -499 |
| Daw1 | chr1 | 83155626 | 25,35695399 | -711 | 83273114 | -21,2244898 | 116777 |
| Dbx2 | chr15 | 95485550 | 21,45061728 | -348 | 95485841 | -21,77644282 | -639 |
| Dmpk | chr7 | 19668556 | 23,20987654 | -642 | 19667466 | -20,94298246 | -1732 |
| Dst | chr1 | 34245672 | -23,20884146 | 28497 | 34234868 | -22,02531646 | 17693 |
| Dst | chr1 | 34246343 | 20,02262443 | 29168 | 34234895 | -21,68560606 | 17720 |
| Dst | chr1 | 34249160 | 21,875 | 31985 | 34246351 | -20,39191672 | 29176 |
| Dst | chr1 | 34248311 | 24,29233145 | 31136 | 34234889 | -20,25974026 | 17714 |
| Dvl1 | chr4 | 155226370 | 24,31338028 | 4849 | 155220873 | -21,64502165 | -648 |
| Epha5 | chr5 | 84846913 | 21,0742019 | -506 | 84847084 | -20,92170725 | -677 |
| Fam101b | chr11 | 75843138 | 23,66603892 | -1854 | 75894628 | -22,61403509 | -53344 |
| Fam83a | chr15 | 57816028 | 21,97474168 | -1430 | 57816394 | -21,07007576 | -1064 |
| Fam83h | chr15 | 75834751 | 21,88697318 | 5177 | 75833084 | -29,05982906 | 6844 |
| Fam83h | chr15 | - | - | - | 75834743 | 21,53846154 | 5185 |
| Fam83h | chr15 | - | - | - | 75834753 | 26,10294118 | 5175 |
| Fndc7 | chr3 | 108684524 | 23,03167421 | 8402 | 108684520 | -22,66139657 | 8406 |
| Gja1 | chr10 | 56096040 | 21,17748918 | -1066 | 56107432 | -20,86247086 | 10326 |
| Gm21637 | chrX | 30822165 | 23,9946973 | -1085 | 31430788 | -24,52867243 | -1091 |
| Gne | chr4 | 44097721 | 22.2222222222222-2 | -672 | 44086351 | -22,3125 | -806 |
| Gpr12 | chr5 | 147395134 | 21,44066492 | 846 | 147395227 | -21,55797101 | 753 |
| Gpr35 | chr1 | 94875486 | 22,75787773 | 6 | 94871937 | -21,43522833 | 13 |
| Gsx2 | chr5 | 75471149 | -21,72792855 | -477 | 75473178 | -23,24561404 | 1552 |
| Gsx2 | chr5 | 75472999 | 20,2764977 | 1373 |  |  |  |
| Gtf2a1l | chr17 | 89093696 | 20,01363017 | 25696 | 89067721 | -23,66718028 | -279 |
| Gtf2a1l | chr17 | 89093700 | 22,44129375 | 25700 |  |  |  |
| Helz2 | chr2 | 180969046 | -21,30378096 | 7686 | 180969980 | -20,13680347 | 6752 |
| Helz2 | chr2 | 180969472 | 25,3655914 | 7260 | 180968883 | 22,27272727 | 7849 |
| Hoxc10 | chr15 | 102796027 | 20,72336266 | -1200 | 102796303 | -23,89277389 | -924 |
| Hoxd9 | chr2 | 74534939 | 25,04743833 | -881 | 74535199 | -23,0928905 | -621 |
| Hoxd9 | chr2 | 74535199 | 25,77700078 | -621 |  |  |  |
| Htr5a | chr5 | 28169053 | 23,55686563 | 566 | 28169352 | -20,30700352 | 865 |
| Il2rb | chr15 | 78312391 | 26,70807453 | 13105 | 78312514 | -20,00771754 | 12982 |
| Islr2 | chr9 | 58046655 | 21,48897614 | 2867 | 58047654 | -21,03825137 | 1868 |
| Kcnip4 | chr5 | 49677579 | 21,14728682 | -681 | 49281570 | -24,26847662 | -774 |
| Kcnip4 | chr5 | 49096571 | 21,81251147 | -105650 |  |  |  |
| Klhl40 | chr9 | 121686661 | 22,6800154 | -64 | 121687985 | -21,18875502 | 1260 |
| Lhcgr | chr17 | 89149697 | 23,5664077 | 41619 | 89149266 | -21,97200243 | 42050 |
| Lhcgr | chr17 | - | - | - | 89149697 | -20,67271353 | 41619 |
| Lmna | chr3 | 88297768 | 20,33362094 | -534 | 88286356 | -22,79661017 | 10878 |
| Ly6g5b | chr17 | 35253452 | 25,23809524 | -1107 | 35253399 | -24,10138249 | -1054 |
| Madd | chr2 | 91020558 | 21,2212677 | -1080 | 91019689 | -22,89915966 | -211 |
| Mcpt9 | chr14 | 56648465 | 21,40105922 | 867 | 56648466 | -21,92982456 | 866 |
| Mir3968 | chr11 | 115310198 | 20,7278481 | -824 | 115309480 | -21,5583508 | -106 |
| Mir3968 | chr11 | 115309911 | 23,55575459 | -537 | 115309504 | -21,42857143 | -130 |
| Mir6366 | chr16 | 18166879 | 24,44444444 | -1616 | 18165986 | -20,81818182 | -723 |
| Mir6366 | chr16 | - | - | - | 18151344 | -20,47769582 | 13919 |
| Mir6366 | chr16 | - | - | - | 18151638 | 21,20220814 | 13625 |
| Mir669a-2 | chr2 | 10422799 | 20,13888889 | -161 | 10405590 | -24,19106317 | -227 |
| Mir6916 | chr10 | 127954804 | 23,81217005 | -5718 | 127945910 | -23,04869097 | 3176 |
| Mir6924 | chr11 | 69697299 | 20,64721064 | -1703 | 69699015 | -21,9841793 | -3419 |
| Mir6924 | chr11 | 69698281 | 20,67901235 | -2685 | 69699988 | -21,0955711 | -4392 |
| Mir6924 | chr11 | 69695528 | 22,71483306 | 68 |  |  |  |
| Mir6998 | chr2 | 31467188 | 22,02816901 | -753 | 31467188 | -21,73160173 | -753 |
| Mroh4 | chr15 | 74467354 | 23,79089616 | -606 | 74467353 | -26,73611111 | -605 |
| Mstn | chr1 | 53117367 | 21,0158655 | -1140 | 53118864 | -20,72765073 | 357 |
| Myo1b | chr1 | 51973451 | 22 | -633 | 51835311 | -25,26132404 | 137507 |
| Nab2 | chr10 | 127094744 | 22,13114754 | 9015 | 127102126 | -34,44444444 | 1633 |
| Nab2 | chr10 | - | - | - | 127102138 | -20,4459203 | 1621 |
| Nrxn2 | chr19 | 6455065 | 21,61422709 | 36327 | 6416851 | -20,26872499 | -1887 |
| Pcdhac2 | chr18 | 37306119 | 21,84210526 | 2496 | 37304851 | -21,19482496 | 1228 |
| Pcdhgc5 | chr18 | 37977680 | 29,33723197 | -1520 | 37977536 | -22,76570048 | -1664 |
| Plch2 | chr4 | 154400000 | 21,72876304 | -14907 | 154386186 | -22 | -1093 |
| Popdc2 | chr16 | 38362934 | 21,57842158 | 638 | 38362035 | -21,28654971 | -224 |
| Rgs22 | chr15 | 36072011 | 22,44402985 | -1856 | 36072011 | -22,85938727 | -1856 |
| Rltpr | chr8 | 108219225 | 23,74470659 | 4419 | 108214665 | -21,38930535 | -141 |
| Rpl3l | chr17 | 24869332 | 23,98861253 | -298 | 24868806 | -20,99286178 | -824 |
| Rtn1 | chr12 | 73464721 | -22,25534029 | 45320 | 73336974 | -22,17653509 | -241 |
| Rtn1 | chr12 | 73405365 | 21,72670097 | -67651 | 73404971 | -20,14589144 | -67257 |
| Scg2 | chr1 | 79431950 | 23,79349046 | 4715 | 79432688 | -21,6608369 | 3977 |
| Schip1 | chr3 | 68376492 | -24,88207547 | 202 | 68027991 | -21,69059011 | 159267 |
| Schip1 | chr3 | 68228924 | -21,38621201 | -43181 | 68296585 | -20,44807966 | -1518 |
| Schip1 | chr3 | 67868740 | 20,04201681 | 16 | 67868401 | 24,75106686 | -323 |
| Schip1 | chr3 | 68271612 | 23,52112676 | -493 |  |  |  |
| Sh3gl3 | chr7 | 89393078 | 20,44513458 | -15340 | 89408092 | -30,12967201 | -326 |
| Sh3gl3 | chr7 | - | - | - | 89408083 | -29,41919192 | -335 |
| Sh3gl3 | chr7 | - | - | - | 89408003 | -21,78508514 | -415 |
| Siah3 | chr14 | 75855750 | 22,55441909 | -39 | 75855753 | -20,17094017 | -36 |
| Siah3 | chr14 | 75854440 | 25,5582459 | -1349 |  |  |  |
| Slc2a12 | chr10 | 22363978 | 20,60377358 | -839 | 22363902 | -20,52158756 | -915 |
| Slfn5os | chr11 | 82774507 | 20,11605416 | -324 | 82774428 | -21,80650038 | -245 |
| Slfn5os | chr11 | 82775050 | 20,57218144 | -867 |  |  |  |
| Sox6 | chr7 | 122989865 | 25,77083333 | -211 | 123174859 | -23,47480106 | -298 |
| Sox6 | chr7 | - | - | - | 122969248 | -20,15655577 | -1024 |
| Spaca4 | chr7 | 52982979 | 21,69753086 | -1793 | 52983171 | -24,62546816 | -1985 |
| Spaca4 | chr7 | - | - | - | 52982031 | 22.1433814533888-2 | -845 |
| Svil | chr18 | 5063492 | 30,3781858 | 16905 | 5063343 | -20,10582011 | 16756 |
| Tac4 | chr11 | 95122937 | 25,33421357 | 94 | 95118744 | -23,14618644 | -4099 |
| Th | chr7 | 150084015 | 20,56140351 | 1856 | 150084041 | -27,13884993 | 1830 |
| Themis3 | chr17 | 67032774 | 28,7608486 | -88812 | 66909034 | -31,63496377 | 34928 |
| Tmem39a | chr16 | 38602636 | 21,05263158 | 39709 | 38609346 | -27,75823473 | 46419 |
| Tnks1bp1 | chr2 | 84903488 | 22,68578879 | 12871 | 84902180 | -23,77819549 | 11563 |
| Tnks1bp1 | chr2 | - | - | - | 84902831 | -21,4391912 | 12214 |
| Tnks1bp1 | chr2 | - | - | - | 84903085 | 22,54901961 | 12468 |
| Tnrc18 | chr5 | 143533592 | 22,37654321 | 45474 | 143521783 | -22,40640715 | 57283 |
| Tnrc18 | chr5 | 143533780 | 23,44180023 | 45286 |  |  |  |
| Tor1aip2 | chr1 | 157912276 | 21,13690092 | 24285 | 157912126 | -22,16450216 | 24135 |
| Tor1aip2 | chr1 | - | - | - | 157909285 | -21,94217939 | 21294 |
| Traf1 | chr2 | 34819118 | 22,12938649 | -1826 | 34818960 | -22,15108835 | -1668 |
| Traf1 | chr2 | 34819154 | 28,31765935 | -1862 |  |  |  |
| Tsc22d1 | chr14 | 76887484 | -21,84035477 | -759 | 76903487 | -26,06060606 | -830 |
| Tsc22d1 | chr14 | 76887813 | 20,71298465 | -430 |  |  |  |
| Ttn | chr2 | 76746280 | 22,69879518 | 74324 | 76880660 | -20,40229885 | -60056 |
| Vwf | chr6 | 125502917 | 24,78448276 | -49 | 125502621 | -20,88235294 | -345 |
| Zak | chr2 | 72123003 | 20,24096386 | -691 | 72049498 | -24,76255088 | -74196 |
| Zfhx2os | chr14 | 55691223 | 20,55749129 | -705 | 55691321 | -20,68181818 | -607 |
| Zfp957 | chr14 | 79613663 | 26,72636816 | 33511 | 79613694 | -20,89552239 | 33480 |
| 1700027F09Rik | chr5 | 64853873 | 21,27594628 | 5308 | 64860049 | -25,67920585 | -868 |
| 2510039O18Rik | chr4 | 147318696 | 24,38423645 | 3692 | 147316111 | -21,71717172 | 1107 |
| 2900052N01Rik | chr9 | 46426703 | 23,60922659 | -294983 | 46427054 | -25,75757576 | -294632 |
| 4931430N09Rik | chr6 | 118708091 | 23,31213307 | -122085 | 118821348 | -20,12867647 | -8828 |
| 4933416C03Rik | chr10 | 115551003 | 20,18444147 | -30 | 115551156 | -20,84880637 | -183 |
| 4933417O13Rik | chr7 | 150516679 | 23,47826087 | -124 | 150517220 | -22,33538819 | -665 |

**Table S8: list of genes hypermethylated in females and hypomethylated in males after GR knockout**

| Gene name | Chr. | Pos. ♂ | Delta beta ♂ | Distance to TSS ♂ | Pos. ♀ | Delta beta ♀ | Distance to TSS ♀ |
| --- | --- | --- | --- | --- | --- | --- | --- |
| Aldh1a7 | chr19 | 20802740 | -25,19355603 | -694 | 20802105 | 22,14046823 | -59 |
| Aldh8a1 | chr10 | 21096536 | -22,0414673 | -570 | 21096548 | 21,42857143 | -558 |
| Alpl | chr4 | 137282838 | -21,52380952 | 39552 | 137353850 | 20,2432825 | -1551 |
| Amigo2 | chr15 | 97214708 | -20,66706373 | -136990 | 97214730 | 20,32786885 | -137012 |
| Amigo2 | chr15 | - | - | - | 97214744 | 20,97222222 | -137026 |
| Amigo2 | chr15 | - | - | - | 97214596 | 21,32107023 | -136878 |
| Amigo2 | chr15 | - | - | - | 97214742 | 21,70868347 | -137024 |
| Amigo2 | chr15 | - | - | - | 97214734 | 22,98912074 | -137016 |
| Amz1 | chr5 | 141198780 | -20,47955975 | -1301 | 141224714 | 22,16216216 | 24633 |
| AU015791 | chr12 | 106750433 | -20,34482759 | -1210 | 106750767 | -20,97601642 | -876 |
| AU015791 | chr12 | - | - | - | 106750424 | 20,68965517 | -1219 |
| B930025P03Rik | chr8 | 10883157 | -27,62908325 | -703 | 10644911 | -21,58528852 | 237543 |
| B930025P03Rik | chr8 | - | - | - | 10883249 | 20,08547009 | -795 |
| B930025P03Rik | chr8 | - | - | - | 10646651 | 26,9858156 | 235803 |
| Babam1 | chr8 | 73921528 | -23,83908046 | 774 | 73921528 | 29,09288599 | 774 |
| Brpf3 | chr17 | 28944134 | -21,03372029 | 6063 | 28954050 | 22,96296296 | 15979 |
| C77080 | chr4 | 128899433 | -25,6980057 | 5722 | 128901138 | -21,9047619 | 4017 |
| C77080 | chr4 | 128917906 | 29,03225806 | -1444 | 128901150 | -21,84628237 | 4005 |
| C77080 | chr4 | - | - | - | 128903171 | 20,11481056 | 1984 |
| Col7a1 | chr9 | 108855816 | -23,45511077 | 26 | 108849040 | 23,10606061 | -6750 |
| Coro2b | chr9 | 62337117 | -20,95840868 | 47734 | 62337117 | 20,95332671 | 47734 |
| Ddx50 | chr10 | 62128358 | -20,63829787 | -14412 | 62128699 | 23,94410924 | -14753 |
| Dusp16 | chr6 | 134744074 | -21,02678571 | -1428 | 134668521 | 34,99511241 | 74125 |
| Ehd2 | chr7 | 16549401 | -22,58297258 | 3483 | 16549378 | 23,14814815 | 3506 |
| Frem1 | chr4 | 82668346 | -31,18908382 | -1340 | 82618191 | 26,16438356 | 48815 |
| Frem1 | chr4 | 82618171 | 25,4978355 | 48835 |  |  |  |
| Gm16853 | chr9 | 21207822 | -23,73626374 | 8342 | 21207822 | 20,10233918 | 8342 |
| Gm16853 | chr9 | 21207827 | -22,52236136 | 8337 | - | - | - |
| Gm1943 | chr8 | 111886916 | -26,47916667 | -22108 | 111837761 | 20,11311417 | 27047 |
| Gm20751 | chr13 | 43830136 | -32,61904762 | 17976 | 43783214 | 22,44268078 | -28946 |
| Gmppa | chr1 | 75423634 | -29,09090909 | -8884 | 75418309 | 20,63519611 | -14209 |
| Gmppa | chr1 | 75418309 | -21,29060579 | -14209 | 75418756 | 22,2345542 | -13762 |
| Gna13 | chr11 | 109222521 | -22,89332138 | -1587 | 109222415 | 20,13264869 | -1693 |
| Gpr39 | chr1 | 127574263 | -20,08097166 | 690 | 127574441 | 22,4137931 | 868 |
| Gpr39 | chr1 | 127573958 | 20,10869565 | 385 | - | - | - |
| Gpr39 | chr1 | 127573858 | 24,70034247 | 285 | - | - | - |
| Helz2 | chr2 | 180969046 | -21,30378096 | 7686 | 180969980 | -20,13680347 | 6752 |
| Helz2 | chr2 | 180969472 | 25,3655914 | 7260 | 180968883 | 22,27272727 | 7849 |
| Itgb7 | chr15 | 102062605 | -35,51948052 | -239 | 102062883 | 21,24968378 | -517 |
| Jup | chr11 | 100244467 | -20,11449388 | 14637 | 100260182 | 23,99062725 | -1078 |
| Kansl1 | chr11 | 104223253 | -24,27140255 | 80352 | 104285544 | 26,11888112 | 18061 |
| Khk | chr5 | 31222519 | -22,90960452 | -1749 | 31223211 | 24,35443038 | -1057 |
| Khk | chr5 | 31222887 | -21,71412137 | -1381 | - | - | - |
| Khk | chr5 | 31223028 | 30,59701493 | -1240 | - | - | - |
| Krt80 | chr15 | 101200444 | -20,65831491 | 112 | 101200465 | 20,90524259 | 91 |
| Krt80 | chr15 | 101200467 | 23,71794872 | 89 | - | - | - |
| Lrrc34 | chr3 | 30545829 | -20,61781609 | 911 | 30547907 | 23,21937322 | -1167 |
| Mab21l2 | chr3 | 86350881 | -20,31778229 | 1324 | 86350904 | 21,36150235 | 1301 |
| Mir365-2 | chr11 | 79538850 | -20,49277422 | -1052 | 79537983 | 22,12750185 | -1919 |
| Mir671 | chr5 | 24097378 | -26,76640231 | -554 | 24096835 | 24,45856874 | -1097 |
| Mir671 | chr5 | 24097382 | -26,47658927 | -550 | - | - | - |
| Mir6933 | chr11 | 117911099 | -21,09090909 | 47515 | 117913938 | 24,57142857 | 50354 |
| Msx2 | chr13 | 53563905 | -20,8168643 | 4244 | 53563866 | 20,56050288 | 4283 |
| Mtl5 | chr19 | 3388285 | -26,73509286 | -572 | 3389596 | 23,82302893 | 218 |
| Nadk | chr4 | 154929271 | -22,24367224 | -7230 | 154946358 | 26,16995074 | 8434 |
| Nptxr | chr15 | 79635920 | -24,125 | -781 | 79636139 | 24,89795918 | -1000 |
| Olfr1394 | chr11 | 48973036 | -29,59839357 | -482 | 48973005 | 20,28301887 | -513 |
| Plch1 | chr3 | 63501470 | -23,13043478 | 153443 | 63501700 | 20,71834416 | 153213 |
| Pou6f1 | chr15 | 100417388 | -20,04357298 | -592 | 100417389 | 20,449521 | -593 |
| Rn4.5s | chr6 | 47624151 | -22,87669195 | -1753 | 47611209 | 22,87644788 | -1795 |
| Rn4.5s | chr6 | 47687452 | -22,34408602 | 6778 | 47687937 | 23,11066127 | 6293 |
| Rn4.5s | chr6 | 47718702 | -20,81280788 | -15778 | 47692180 | 23,90678188 | 2050 |
| Rn4.5s | chr6 | 47614938 | 20,67873303 | -1184 | 47603601 | 27,68764486 | 1492 |
| Rn4.5s | chr6 | 47598749 | 22,25274725 | 6344 | - | - | - |
| Rn4.5s | chr6 | 47704338 | 22,97607342 | -1414 | - | - | - |
| Rn4.5s | chr6 | 47715156 | 24,31818182 | -12232 | - | - | - |
| Rn4.5s | chr6 | 47633865 | 22,02 | 1510 | - | - | - |
| Rp1l1 | chr14 | 64610657 | -23,14814815 | -611 | 64611415 | 22,06208426 | 147 |
| Rtfdc1 | chr2 | 172252678 | -21,16402116 | -13400 | 172253210 | 20,50865801 | -12868 |
| Rtfdc1 | chr2 | - | - | - | 172253357 | 21,98830409 | -12721 |
| Rusc1 | chr3 | 88891797 | -25,92592593 | 2156 | 88891797 | -30,85255066 | 2156 |
| Rusc1 | chr3 | - | - | - | 88891798 | 21.2121212121212-2 | 2155 |
| Schip1 | chr3 | 68376492 | -24,88207547 | 202 | 68027991 | -21,69059011 | 159267 |
| Schip1 | chr3 | 68228924 | -21,38621201 | -43181 | 68296585 | -20,44807966 | -1518 |
| Schip1 | chr3 | 67868740 | 20,04201681 | 16 | 67868401 | 24,75106686 | -323 |
| Schip1 | chr3 | 68271612 | 23,52112676 | -493 | - | - | - |
| Slc14a2 | chr18 | 78403681 | -22,1875 | -502 | 78403707 | 21,43420016 | -528 |
| Slc14a2 | chr18 | 78403707 | -22,16248507 | -528 | 78403712 | 23,16176471 | -533 |
| Slc38a8 | chr8 | 122026536 | -24,81527094 | -938 | 122026459 | 22,2038111 | -861 |
| Smarcal1 | chr1 | 72632734 | -20,81632653 | 2909 | 72637771 | 21,67253521 | 7946 |
| Tcam1 | chr11 | 106147790 | -21,67046565 | 9804 | 106136440 | -23,83126767 | -1546 |
| Tcam1 | chr11 | - | - | - | 106137581 | 27,57885763 | -405 |
| Tead3 | chr17 | 28478403 | -25,47169811 | 8888 | 28489128 | 21,17244158 | -1378 |
| Tiam2 | chr17 | 3483231 | -28,33333333 | -259 | 3414182 | -24,71794872 | 16975 |
| Tiam2 | chr17 | 3397109 | -20,75949367 | -98 | 3483336 | 20,95238095 | -154 |
| Tiam2 | chr17 | - | - | - | 3483205 | 22,23707665 | -285 |
| Vav2 | chr2 | 27283250 | -25,88235294 | -905 | 27283264 | 21,22475856 | -919 |
| 8430437L04Rik | chr2 | 72541629 | -20,6622879 | 62 | 72541519 | 25,55147059 | -48 |

**Table S9: CGs methylated in the same location in both sexes**

| Gene name | Annotation | Chr. | Location | Distance to TSS | Nearest ENSEMBL |
| --- | --- | --- | --- | --- | --- |
| Babam1 | intron (NM_026636, intron 1 of 8) | 8 | 73921528 | 774 | ENSMUSG00000031820 |
| Cacnb1 | exon (NM_031173, exon 2 of 13) | 11 | 97880999 | -1313 | ENSMUSG00000020882 |
| Coro2b | exon (NM_175484, exon 2 of 12) | 9 | 62337117 | 47734 | ENSMUSG00000041729 |
| Fshr | Intergenic | 17 | 89601266 | -1251 | ENSMUSG00000032937 |
| Gmppa | exon (NM_007463, exon 30 of 41) | 1 | 75418309 | -14209 | ENSMUSG00000033021 |
| Gm16853 | exon (NM_010561, exon 19 of 21) | 9 | 21207822 | 8342 | ENSMUSG00000097665 |
| Hoxd9 | promoter-TSS (NM_013555) | 2 | 74535199 | -621 | ENSMUSG00000043342 |
| Lhcgr | intron (NM_013582, intron 9 of 10) | 17 | 89149697 | 41619 | ENSMUSG00000024107 |
| Mir6998 | promoter-TSS (NR_105964) | 2 | 31467188 | -753 | - |
| Mir7078 | intron (NM_028941, intron 20 of 20) | 8 | 119981505 | -1660 | - |
| Paqr4 | promoter-TSS (NM_023824) | 17 | 23878144 | -847 | ENSMUSG00000023909 |
| Rgs22 | Intergenic | 15 | 36072011 | -1856 | ENSMUSG00000037627 |
| Rusc1 | exon (NM_001083808, exon 7 of 9) | 3 | 88891797 | 2156 | ENSMUSG00000041263 |
| Slc14a2 | promoter-TSS (NM_001110274) | 18 | 78403707 | -528 | ENSMUSG00000024552 |
| Sumo2 | promoter-TSS (NM_133354) | 11 | 115398391 | -847 | ENSMUSG00000020738 |
| 4930487D11Rik | promoter-TSS (NR_046191) | 5 | 38735225 | -403 | - |
| 5033406O09Rik | Intergenic | 12 | 113184383 | -1690 | - |

**Table S10: Candidate genes: ANOVA and mediation/moderation analysis; - : “not significant”.**

| **Cand. genes** | ANOVA (with average methylation level of CGs) | Mediation/moderation |
| --- | --- | --- |
| Ank3 | - | - |
| Avp | - | - |
| Avpr1a | - | - |
| Avpr1b | - | - |
| Bdnf | - | - |
| Cacna1c | sex*genotype: F_3,8_=5.689, p=0.044 | - |
| Cyp11b1 | - | - |
| Cyp11b2 | - | - |
| Fkbp5 | genotype: F_3,8_=10.537, p=0.012, lower methylation in GR^+/-^, only significant in females (t_4_=-6.094, p=0.004) | moderation: F_1,8_=18.1987, p=0.0027 |
| Hsd11b1 | sex*genotype: F_3,8_=32.883, p<0.001, significantly more methylation in GR^+/-^ males compared to GR^+/-^ females (t_4_=6.007, p=0.004) and wildtype males (t_4_=5.853, p=0.004) | - |
| Igf2 | sex: F_3,8_=25.378, p=0.001, lower methyl. in ♀,  only significant in wildtypes (t_2.203_=6.143, p=0.004) | - |
| Morc1 | - | - |
| Nr3c1 | - | - |
| Oxt | - | - |
| Oxtr | - | - |
| Pclo | - | - |
| Slc6a4 | sex*genotype: F_3,8_=5.197, p=0.052 | - |

**Table S11: Selection of molecules in networks with basal metabolic and neuronal functions associated with genes differentially methylated in both sexes**

| **Gene abbreviation** | **Gene name** | **Number of networks** |
| --- | --- | --- |
| ADRA2A | adrenoceptor alpha 2A | 3 |
| ADRB2 | adrenoceptor beta 2, surface | 2 |
| AGTR1 | angiotensin II receptor, type 1 | 2 |
| APBB1 | amyloid beta (A4) precursor protein-binding, family B, member 1 (Fe65) | 4 |
| APOE | apolipoprotein E | 4 |
| ARRB1 | arrestin, beta 1 | 2 |
| CNR1 | cannabinoid receptor 1 (brain) | 4 |
| CREB3 | cAMP responsive element binding protein 3 | 1 |
| CREBBP | CREB binding protein | 5 |
| D2HGDH | D-2-hydroxyglutarate dehydrogenase |  |
| DRD5 | dopamine receptor D5 | 4 |
| HSD11B1 | hydroxysteroid (11-beta) dehydrogenase 1 | 4 |
| HSD17B3 | hydroxysteroid (17-beta) dehydrogenase 3 | 4 |
| HTT | huntingtin | 4 |
| LIPE | lipase, hormone-sensitive | 2 |
| LPL | lipoprotein lipase | 4 |
| MAPT | microtubule-associated protein tau | 2 |
| MARK2 | MAP/microtubule affinity-regulating kinase 2 | 3 |
| NOS1 | nitric oxide synthase 1 (neuronal) | 1,7 |
| NR1H3 | nuclear receptor subfamily 1, group H, member 3 | 4 |
| PCDH9 | protocadherin 9 | 5 |
| PDK4 | pyruvate dehydrogenase kinase, isozyme 4 | 1 |
| PGR | progesterone receptor | 4 |
| POMC | proopiomelanocortin | 4 |
| PPARD | peroxisome proliferator-activated receptor delta | 1 |
| PPARG | peroxisome proliferator-activated receptor gamma | 2 |
| PYGL | phosphorylase, glycogen, liver | 2 |
| RASD1 | RAS, dexamethasone-induced 1 | 7 |
| ROCK2 | Rho-associated, coiled-coil containing protein kinase 2 |  |
| RTN1 | reticulon 1 | 4 |
| SCARB1 | scavenger receptor class B, member 1 | 4 |
| SCNN1A | sodium channel, non voltage gated 1 alpha subunit | 3 |
| SCNN1B | sodium channel, non voltage gated 1 beta subunit | 3 |
| SCNN1G | sodium channel, non voltage gated 1 gamma subunit | 3 |
| SGK1 | serum/glucocorticoid regulated kinase 1 |  |
| SGPL1 | sphingosine-1-phosphate lyase 1 | 1 |
| YWHAB | tyrosine 3-monooxygenase/tryptophan 5-monooxygenase activation protein, beta |  |
| ZFYVE27 | zinc finger, FYVE domain containing 27 | 4 |

**Table S12: Upstream regulators associated with genes differentially methylated in both sexes**

| **Upstream regulator** | **Molecule type** | **p-value of overlap** |
| --- | --- | --- |
| CNR1 | g-protein coupled receptor | 3.81E-03 |
| TNFSF12 | cytokine | 4.26E-03 |
| Cebp | complex | 5.43E-03 |
| CYP11B1 | enzyme | 5.43E-03 |
| YWHAB | transcription regulator | 5.43E-03 |
| ESR2 | ligand-dependent nuclear receptor | 5.99E-03 |
| Ifi202b | other | 1.08E-02 |
| MLLT3 | other | 1.08E-02 |
| ALKBH5 | enzyme | 1.11E-02 |
| CREBBP | transcription regulator | 1.34E-02 |
| IFNG | cytokine | 1.44E-02 |
| PPIF | enzyme | 1.50E-02 |
| TMEM27 | other | 1.62E-02 |
| MYF6 | transcription regulator | 1.62E-02 |
| mir-25 | microrna | 1.62E-02 |
| TRPC1 | ion channel | 1.62E-02 |
| TBC1D4 | other | 1.62E-02 |
| EPC1 | transcription regulator | 2.16E-02 |
| PSMB9 | peptidase | 2.69E-02 |
| mir-19 | microrna | 2.69E-02 |
| SLC12A2 | transporter | 2.69E-02 |
| P2RY2 | g-protein coupled receptor | 2.69E-02 |
| SOX15 | transcription regulator | 2.69E-02 |
| STAU1 | transporter | 2.69E-02 |
| DBP | transcription regulator | 2.69E-02 |
| SGK1 | kinase | 2.69E-02 |
| HLF | transcription regulator | 2.69E-02 |
| ARHGDIA | other | 2.69E-02 |
| BIRC3 | enzyme | 3.22E-02 |
| DOT1L | phosphatase | 3.22E-02 |
| ELAVL1 | other | 3.22E-02 |
| PSMB10 | peptidase | 3.74E-02 |
| ROCK2 | kinase | 3.74E-02 |
| mir-17 | microrna | 3.74E-02 |
| TEF | transcription regulator | 3.74E-02 |
| TNFRSF12A | transmembrane receptor | 3.74E-02 |
| NLRP3 | other | 4.09E-02 |
| KCNE3 | ion channel | 4.22E-02 |
| TTN | kinase | 4.26E-02 |
| PSMB8 | peptidase | 4.26E-02 |
| TDGF1 | growth factor | 4.26E-02 |
| NCKAP1L | other | 4.78E-02 |
| FGFR4 | kinase | 4.78E-02 |
| TNFRSF13C | transmembrane receptor | 4.78E-02 |
| ADAM12 | peptidase | 4.78E-02 |
| Hmga2 | enzyme | 4.78E-02 |

**Table S13: list of genes in GR+/- fetal placental tissue overlapping with GR+/- frontal cortex tissue**

| Gene name | Delta beta | Chromosome | position | Distance to TSS |
| --- | --- | --- | --- | --- |
| Itgb7 | -35,51948052 | chr15 | 102062605 | -239 |
| Papln | -29,75423046 | chr12 | 85103234 | -1350 |
| Haao | -29,62577963 | chr17 | 84247524 | -1394 |
| Inha | -29,44444444 | chr1 | 75504610 | 958 |
| Gmppa | -29,09090909 | chr1 | 75423634 | -8884 |
| Tiam2 | -28,33333333 | chr17 | 3483231 | -259 |
| Dpp6 | -27,77777778 | chr5 | 27143010 | -887 |
| B930025P03Rik | -27,62908325 | chr8 | 10883157 | -703 |
| Mir671 | -26,76640231 | chr5 | 24097378 | -554 |
| Gm1943 | -26,47916667 | chr8 | 111886916 | -22108 |
| Mir671 | -26,47658927 | chr5 | 24097382 | -550 |
| Tiam1 | -26,36363636 | chr16 | 89818786 | -189 |
| A330009N23Rik | -26,08695652 | chr15 | 101062009 | -6392 |
| A330009N23Rik | -25,81967213 | chr15 | 101062027 | -6410 |
| Narg2 | -25,76800847 | chr9 | 69212085 | -33720 |
| Gca | -25,7020757 | chr2 | 62553098 | 50714 |
| Dclk3 | -25,62468556 | chr9 | 111370560 | 28975 |
| Ubxn10 | -25,61146052 | chr4 | 138294566 | -213 |
| Lrrc32 | -25,28467909 | chr7 | 105641246 | -1486 |
| Mettl24 | -25,22058824 | chr10 | 40444175 | 41087 |
| Cog2 | -25,14814815 | chr8 | 127043951 | -716 |
| Ino80e | -25,12779123 | chr7 | 134004484 | 493 |
| Hes3 | -25,04957039 | chr4 | 151666713 | -942 |
| Nr3c1 | -24,69135802 | chr18 | 39588381 | 58518 |
| Olfr161 | -24,66931217 | chr16 | 3592561 | 163 |
| Anks1b | -24,57431154 | chr10 | 90039167 | -269 |
| Cdkal1 | -24,47916667 | chr13 | 30010500 | -63043 |
| Prima1 | -24,45436508 | chr12 | 104481972 | -1616 |
| Mical3 | -24,39668175 | chr6 | 121031887 | -242 |
| Ccdc33 | -24,32258065 | chr9 | 57965021 | 1609 |
| Nptxr | -24,125 | chr15 | 79635920 | -781 |
| Map3k14 | -24,09502262 | chr11 | 103129882 | -1167 |
| Sez6 | -24,02380952 | chr11 | 77767115 | 22670 |
| Itgad | -24,00488028 | chr7 | 135317457 | -3 |
| Macrod1 | -23,875 | chr19 | 7130691 | -567 |
| Nin | -23,81818182 | chr12 | 71205298 | -1457 |
| Lag3 | -23,81733021 | chr6 | 124860364 | 1359 |
| Mir101c | -23,7199582 | chr9 | 3017398 | 21345 |
| Tgfbi | -23,3734281 | chr13 | 56729350 | 18386 |
| Cul9 | -23,3572282 | chr17 | 46663654 | 19683 |
| Thtpa | -23,23733863 | chr14 | 55712706 | -915 |
| G530011O06Rik | -23,21428571 | chrX | 166425238 | -8389 |
| Dst | -23,20884146 | chr1 | 34245672 | 28497 |
| Kcnf1 | -23,18313953 | chr12 | 17182075 | 1619 |
| Gm17019 | -23,16479401 | chr5 | 15054864 | -21857 |
| Plch1 | -23,13043478 | chr3 | 63501470 | 153443 |
| Coro1c | -23,02083333 | chr5 | 114393286 | -34571 |
| Myl12b | -23,01392301 | chr17 | 71341196 | -1340 |
| Fam58b | -23,00984529 | chr11 | 78566183 | -952 |
| Mirlet7i | -22,90037831 | chr10 | 122450445 | -27665 |
| Rn4.5s | -22,87669195 | chr6 | 47624151 | -1753 |
| Oas1h | -22,84482759 | chr5 | 121311634 | 203 |
| Gm19303 | -22,84334023 | chr15 | 51227611 | -455 |
| D130017N08Rik | -22,74193548 | chr5 | 144519047 | -176 |
| Cacnb3 | -22,55291005 | chr15 | 98469944 | 4746 |
| Pde6h | -22,51626257 | chr6 | 136928254 | 25210 |
| Mir684-1 | -22,42647059 | chr16 | 20177171 | -864 |
| Abcc3 | -22,4137931 | chr11 | 94270646 | -16356 |
| Rn4.5s | -22,34408602 | chr6 | 47687452 | 6778 |
| Dyx1c1 | -22,33952703 | chr9 | 72805718 | -874 |
| Tgm6 | -22,33589088 | chr2 | 129937710 | -11311 |
| Mgst2 | -22,3092999 | chr3 | 51655977 | 190862 |
| Stpg2 | -22,28158391 | chr3 | 138868110 | -747 |
| Rtn1 | -22,25534029 | chr12 | 73464721 | 45320 |
| Nadk | -22,24367224 | chr4 | 154929271 | -7230 |
| A330035P11Rik | -22,20563847 | chr14 | 122505039 | 1157 |
| Slc14a2 | -22,1875 | chr18 | 78403681 | -502 |
| Slc14a2 | -22,16248507 | chr18 | 78403707 | -528 |
| Pbx3 | -22,10526316 | chr2 | 34229107 | -1551 |
| Muc6 | -22,08643815 | chr7 | 148824045 | 17162 |
| Acads | -22,04433498 | chr5 | 115567496 | 1859 |
| Sprr2b | -21,99134199 | chr3 | 92120259 | -368 |
| Gm4559 | -21,84551887 | chr7 | 149467393 | -7125 |
| Srcin1 | -21,81020734 | chr11 | 97413093 | 23347 |
| Sertad3 | -21,74636175 | chr7 | 28261291 | 2432 |
| Kcnab2 | -21,69717961 | chr4 | 151823418 | -454 |
| Rnf165 | -21,67504769 | chr18 | 77705730 | 98145 |
| Asphd2 | -21,4951715 | chr5 | 112821621 | -388 |
| Gm19303 | -21,42022209 | chr15 | 51227620 | -464 |
| Schip1 | -21,38621201 | chr3 | 68228924 | -43181 |
| A2m | -21,38047138 | chr6 | 121682817 | 96626 |
| Htt | -21,33333333 | chr5 | 35086091 | -18298 |
| Gmppa | -21,29060579 | chr1 | 75418309 | -14209 |
| Capn8 | -21,24094203 | chr1 | 184494643 | -495 |
| Gm5072 | -21,2244898 | chrX | 88724494 | -971 |
| Cul9 | -21,21212121 | chr17 | 46658783 | 24554 |
| Aqp3 | -21,14183764 | chr4 | 41046367 | -1151 |
| A330009N23Rik | -21,06060606 | chr15 | 101062012 | -6395 |
| Tgif2lx2 | -20,84577114 | chrX | 115595361 | -1015 |
| Rn4.5s | -20,81280788 | chr6 | 47718702 | -15778 |
| Tiam2 | -20,75949367 | chr17 | 3397109 | -98 |
| Ddx50 | -20,63829787 | chr10 | 62128358 | -14412 |
| Sorbs3 | -20,625 | chr14 | 70593389 | -1336 |
| 4930505G20Rik | -20,57471264 | chr14 | 115809515 | -741 |
| Lilra5 | -20,52360916 | chr7 | 4189298 | -58 |
| Htatip2 | -20,5028368 | chr7 | 56997268 | -17208 |
| Ctif | -20,4966642 | chr18 | 75858093 | -743 |
| Mir365-2 | -20,49277422 | chr11 | 79538850 | -1052 |
| Fgfr2 | -20,47930283 | chr7 | 137386063 | 24259 |
| Ptpro | -20,47619048 | chr6 | 137192489 | -8331 |
| Gm13283 | -20,42954426 | chr4 | 88405501 | -1177 |
| Oxr1 | -20,42253521 | chr15 | 40927630 | -351398 |
| Ptk2b | -20,37815126 | chr14 | 66801326 | 31063 |
| Apbb2 | -20,35973976 | chr5 | 67011188 | -1132 |
| AU015791 | -20,34482759 | chr12 | 106750433 | -1210 |
| Dclk3 | -20,31947784 | chr9 | 111370721 | 29136 |
| Fzd5 | -20,31746032 | chr1 | 64782289 | 2035 |
| A330035P11Rik | -20,25016903 | chr14 | 122505662 | 534 |
| Jup | -20,11449388 | chr11 | 100244467 | 14637 |
| Olfr161 | -20,10347376 | chr16 | 3592105 | -293 |
| Pde2a | -20,07389163 | chr7 | 108569458 | -747 |
| Ap3m1 | -20,06462985 | chr14 | 21826424 | 45240 |
| Pitpnm2 | -20,04479283 | chr5 | 124637670 | 28757 |
| Adsl | 20 | chr15 | 80778172 | -748 |
| Dst | 20,02262443 | chr1 | 34246343 | 29168 |
| Mnd1-ps | 20,02713704 | chr14 | 10715838 | -771 |
| Scn8a | 20,0304878 | chr15 | 100766630 | -72 |
| Schip1 | 20,04201681 | chr3 | 67868740 | 16 |
| Cntn1 | 20,08387698 | chr15 | 91991507 | -281 |
| Plxnd1 | 20,08797654 | chr6 | 115930636 | 14387 |
| Asphd2 | 20,09397457 | chr5 | 112820615 | 618 |
| Gucy1b3 | 20,09456265 | chr3 | 81879256 | -623 |
| Zfp366 | 20,11251758 | chr13 | 99954968 | 190 |
| Grik3 | 20,13824885 | chr4 | 125166068 | -2007 |
| Tacc2 | 20,15475313 | chr7 | 137834240 | -1830 |
| Qrfp | 20,17057569 | chr2 | 31666530 | -492 |
| Slc7a4 | 20,21978022 | chr16 | 17575706 | 1058 |
| Mir7656 | 20,23076923 | chr9 | 94652015 | -233 |
| Ascl1 | 20,23079697 | chr10 | 86940776 | 15629 |
| Zak | 20,24096386 | chr2 | 72123003 | -691 |
| Acr | 20,25641026 | chr15 | 89398573 | -184 |
| Mir144 | 20,27715833 | chr11 | 77885581 | -926 |
| 1700029N11Rik | 20,28985507 | chr13 | 44534322 | -774 |
| Tcof1 | 20,30651341 | chr18 | 60999147 | 9471 |
| Ank1 | 20,31312726 | chr8 | 24206142 | 37411 |
| Kcnn3 | 20,31615925 | chr3 | 89323739 | -347 |
| Tox3 | 20,35603715 | chr8 | 92872992 | -841 |
| Swap70 | 20,40816327 | chr7 | 117422803 | 57586 |
| Ubxn10 | 20,40935673 | chr4 | 138294260 | 93 |
| Scaper | 20,42207792 | chr9 | 55786822 | -900 |
| Fut8 | 20,49187527 | chr12 | 78659192 | 148064 |
| Rasl11b | 20,55727554 | chr5 | 74590464 | -887 |
| Srcin1 | 20,59925094 | chr11 | 97438136 | -1696 |
| Slc2a12 | 20,60377358 | chr10 | 22363978 | -839 |
| Haao | 20,64220183 | chr17 | 84257976 | -11846 |
| Rn4.5s | 20,67873303 | chr6 | 47614938 | -1184 |
| Gas7 | 20,68151147 | chr11 | 67409686 | -371 |
| 9530052E02Rik | 20,70707071 | chr8 | 11005631 | -2219 |
| Apol8 | 20,72189606 | chr15 | 77613873 | -28214 |
| Abcc3 | 20,77922078 | chr11 | 94277338 | -23048 |
| Ppp1r15a | 20,78037904 | chr7 | 52779465 | 2173 |
| H1foo | 20,8045977 | chr6 | 115917766 | 22809 |
| Dab2ip | 20,80804578 | chr2 | 35550711 | 3197 |
| Pklr | 20,84861184 | chr3 | 88940122 | 58 |
| 1700125H03Rik | 20,87420043 | chr8 | 70854988 | -37370 |
| Cngb3 | 20,90497738 | chr4 | 19207983 | -14 |
| Arhgef28 | 20,90909091 | chr13 | 98723737 | 252383 |
| Zfp536 | 20,91897 | chr7 | 38353810 | 200961 |
| Smox | 20,9494324 | chr2 | 131350500 | 32902 |
| Mchr1 | 20,95308278 | chr15 | 81068187 | 2258 |
| Tmem81 | 20,95734127 | chr1 | 134419938 | 17131 |
| Fam53b | 20,98765432 | chr7 | 139951503 | 27115 |
| Smox | 21,05532787 | chr2 | 131350539 | 32941 |
| Myo16 | 21,05769231 | chr8 | 10153546 | -377 |
| Agpat4 | 21,0978836 | chr17 | 12358146 | 45996 |
| Cacnb4 | 21,09969559 | chr2 | 52415146 | -1015 |
| Jarid2 | 21,13943028 | chr13 | 44891340 | 64700 |
| Tmem80 | 21,19006849 | chr7 | 148515178 | 1149 |
| Dlg1 | 21,22653978 | chr16 | 31754511 | 75 |
| Mir1936 | 21,23287671 | chr12 | 103923298 | -68 |
| 1700027F09Rik | 21,27594628 | chr5 | 64853873 | 5308 |
| 6820431F20Rik | 21,28760038 | chr8 | 20010804 | 9474 |
| Rgs7bp | 21,28879892 | chr13 | 105752184 | 92826 |
| Dab2ip | 21,2900382 | chr2 | 35545721 | -1793 |
| Timp4 | 21,34914752 | chr6 | 115202971 | -1104 |
| Ank1 | 21,39406487 | chr8 | 24144319 | -1252 |
| Thap4 | 21,3948319 | chr1 | 95647079 | 4336 |
| Fam53b | 21,4198783 | chr7 | 139951517 | 27101 |
| Mir3102 | 21,42560783 | chr7 | 108032066 | -1143 |
| Slco3a1 | 21,42857143 | chr7 | 81649225 | 50441 |
| Atxn1 | 21,51100376 | chr13 | 46046009 | 14351 |
| H1foo | 21,54471545 | chr6 | 115912726 | 17769 |
| Ablim1 | 21,55720339 | chr19 | 57273052 | -1070 |
| Popdc2 | 21,57842158 | chr16 | 38362934 | 638 |
| Caln1 | 21,60894661 | chr5 | 130809631 | -35697 |
| Mir1952 | 21,67309547 | chr2 | 138645253 | -412 |
| Rtn1 | 21,72670097 | chr12 | 73405365 | -67651 |
| Plch2 | 21,72876304 | chr4 | 154400000 | -14907 |
| Rap2a | 21,76470588 | chr14 | 120784650 | -93033 |
| Dst | 21,875 | chr1 | 34249160 | 31985 |
| Abcd2 | 21,93627451 | chr15 | 91021618 | 620 |
| Hsfy2 | 21,97963801 | chr1 | 56694807 | -512 |
| Ampd3 | 22,00577201 | chr7 | 117917943 | 1825 |
| Rn4.5s | 22,02 | chr6 | 47633865 | 1510 |
| Mir124a-3 | 22,03009207 | chr2 | 180596340 | -32405 |
| Tbc1d30 | 22,04531537 | chr10 | 120748837 | -592 |
| Ccdc167 | 22,06451613 | chr17 | 29827337 | 26625 |
| Npcd | 22,22222222 | chr15 | 79658761 | 6002 |
| Shank2 | 22,22507049 | chr7 | 151360067 | -1358 |
| Rn4.5s | 22,25274725 | chr6 | 47598749 | 6344 |
| Crocc | 22,27602906 | chr4 | 140602929 | 6647 |
| Dcp1a | 22,29957332 | chr14 | 31291942 | -809 |
| Gjb5 | 22,31884058 | chr4 | 127257860 | -222452 |
| Smarcd3 | 22,33766234 | chr5 | 24109396 | -1576 |
| Wdfy3 | 22,37762238 | chr5 | 102366537 | 132403 |
| Dlgap2 | 22,42063492 | chr8 | 14314188 | 218313 |
| Rapgef1 | 22,44664634 | chr2 | 29473998 | -1242 |
| Mir7004 | 22,55442671 | chr2 | 168467334 | -1075 |
| Gpr26 | 22,58823529 | chr7 | 139184444 | 26301 |
| 4933416M06Rik | 22,67958031 | chr13 | 102024207 | -67 |
| Ubxn10 | 22,69585253 | chr4 | 138279670 | 1101 |
| Fam105b | 22,71794872 | chr15 | 27558317 | 2131 |
| F830045P16Rik | 22,7459578 | chr2 | 129363925 | -1587 |
| Hibadh | 22,75550536 | chr6 | 52592082 | -1788 |
| Trim43b | 22,80348759 | chr9 | 88962199 | 25474 |
| Rpl31 | 22,80701754 | chr1 | 39450990 | 26294 |
| Adig | 22,89204098 | chr2 | 158327258 | -1090 |
| Rn4.5s | 22,97607342 | chr6 | 47704338 | -1414 |
| Dpp6 | 23,00204918 | chr5 | 27143519 | -378 |
| Hivep1 | 23,03030303 | chr13 | 42253229 | 105839 |
| G530011O06Rik | 23,08992563 | chrX | 166439209 | -22360 |
| Pcnx | 23,24675325 | chr12 | 83020299 | 59282 |
| 4931430N09Rik | 23,31213307 | chr6 | 118708091 | -122085 |
| 2810029C07Rik | 23,4231731 | chr12 | 112810821 | 1792 |
| Schip1 | 23,52112676 | chr3 | 68271612 | -493 |
| Gm5434 | 23,54357246 | chr12 | 36816677 | -289 |
| Cd160 | 23,76924745 | chr3 | 96632600 | 674 |
| Ang4 | 23,77377377 | chr14 | 52393671 | -406 |
| Dlgap2 | 23,85927505 | chr8 | 14314193 | 218318 |
| Gm3415 | 23,92241379 | chr5 | 147366734 | -955 |
| Tcf12 | 23,967176 | chr9 | 71960609 | -983 |
| Mir1956 | 24,03261675 | chr3 | 138288640 | 99255 |
| Marveld3 | 24,14679757 | chr8 | 112487512 | -1407 |
| Klhl23 | 24,14965986 | chr2 | 69660129 | -298 |
| Cntn4 | 24,16666667 | chr6 | 106068515 | -244 |
| Prodh2 | 24,2114237 | chr7 | 31278835 | 158 |
| Dst | 24,29233145 | chr1 | 34248311 | 31136 |
| Rn4.5s | 24,31818182 | chr6 | 47715156 | -12232 |
| 1600002K03Rik | 24,52380952 | chr10 | 79634878 | -811 |
| Elfn1 | 24,79949875 | chr5 | 140382946 | -951 |
| Art1 | 24,87562189 | chr7 | 109255840 | 5583 |
| Mrvi1 | 24,9475891 | chr7 | 118140230 | -14255 |
| Ryr3 | 25,03706449 | chr2 | 112671339 | 199149 |
| Gal3st1 | 25,22321429 | chr11 | 3883236 | -403 |
| Mri1 | 25,29239766 | chr8 | 86781743 | -520 |
| Gal3st1 | 25,37313433 | chr11 | 3883219 | -420 |
| Mir218-2 | 25,39594332 | chr11 | 35430415 | 97 |
| Npl | 25,41544477 | chr1 | 155390901 | 5943 |
| Snord116l2 | 25,5 | chr7 | 67003189 | 1002 |
| Tead1 | 25,5259467 | chr7 | 119902991 | 93 |
| Sox6 | 25,77083333 | chr7 | 122989865 | -211 |
| Slc39a10 | 25,77652279 | chr1 | 47060923 | -150569 |
| Fhod3 | 25,96656217 | chr18 | 24866870 | -1076 |
| Fos | 26,03836841 | chr12 | 86817279 | 2428 |
| Ptprtos | 26,15184945 | chr2 | 162215709 | -840 |
| Sipa1l2 | 26,28689087 | chr8 | 128016693 | -83 |
| Mt4 | 26,29340893 | chr8 | 96659614 | -1490 |
| Zfp957 | 26,72636816 | chr14 | 79613663 | 33511 |
| Fam53b | 26,76470588 | chr7 | 139978652 | -34 |
| Afap1l2 | 26,81992337 | chr19 | 57024682 | 58383 |
| Enox1 | 27,15846995 | chr14 | 77555092 | -1478 |
| Fstl1 | 27,27272727 | chr16 | 37776278 | -863 |
| Dcaf15 | 27,69567597 | chr8 | 86625660 | 3001 |
| Edn2 | 27,82658518 | chr4 | 119833378 | -651 |
| Prss8 | 28,71572872 | chr7 | 135074408 | -781 |
| Atp6v0d1 | 29,30077691 | chr8 | 108090981 | -1041 |
| Nfkbia | 29,70979638 | chr12 | 56591637 | 1997 |
| Pde6h | 29,83344437 | chr6 | 136928290 | 25246 |
| Mprip | 30,0645682 | chr11 | 59474336 | -1661 |
| Trim46 | 30,45397226 | chr3 | 89048391 | 730 |
| Cog2 | 31,53126826 | chr8 | 127043905 | -762 |
| 2810428I15Rik | 32,13742118 | chr8 | 73031709 | -1071 |

**Table S14: list of molecules in networks with central nervous functions associated with genes differentially methylated in frontal cortex and fetal placental tissue**

| **Gene abbreviation** | **Gene name** | **Number of networks** |
| --- | --- | --- |
| ADSL | adenylosuccinate lyase | 8 |
| APBB2 | amyloid beta (A4) precursor protein-binding, family B, member 2 | 3 |
| APP | amyloid beta (A4) precursor protein |  |
| AP3B2 | adaptor-related protein complex 3, beta 2 subunit | 8 |
| AP3M1 | adaptor-related protein complex 3, mu 1 subunit | 8 |
| AP3S2 | adaptor-related protein complex 3, sigma 2 subunit | 8 |
| ATN1 | atrophin 1 | 2 |
| ATXN1 | ataxin 1 |  |
| BDNF | brain-derived neurotrophic factor | 12 |
| Cacna1 | Calcium Voltage-Gated Channel Subunit Alpha1 A |  |
| CACNA1D | calcium channel, voltage-dependent, L type, alpha 1D subunit | 12 |
| Cacna2d | -- | 12 |
| CACNA2D1 | calcium channel, voltage-dependent, alpha 2/delta subunit 1 | 12 |
| Cacnb | -- | 12 |
| CACNB1 | calcium channel, voltage-dependent, beta 1 subunit | 12 |
| CACNB2 | calcium channel, voltage-dependent, beta 2 subunit | 12 |
| CACNB3 | calcium channel, voltage-dependent, beta 3 subunit | 12 |
| CACNB4 | calcium channel, voltage-dependent, beta 4 subunit | 12 |
| CASK | calcium/calmodulin-dependent serine protein kinase (MAGUK family) |  |
| CNR1 | cannabinoid receptor 1 (brain) |  |
| CNTF | ciliary neurotrophic factor | 2 |
| CNTN1 | contactin 1 | 7 |
| CNTN4 | contactin 4 |  |
| CREBBP | CREB binding protein |  |
| DCLK3 | doublecortin-like kinase 3 | 7 |
| DRD3 | dopamine receptor D3 | 10 |
| DYX1C1 | dyslexia susceptibility 1 candidate 1 | 8 |
| GABRG2 | gamma-aminobutyric acid (GABA) A receptor, gamma 2 |  |
| GAD1 | glutamate decarboxylase 1 (brain, 67kDa) |  |
| GNAO1 | guanine nucleotide binding protein (G protein), alpha activating activity polypeptide O |  |
| GNAQ | guanine nucleotide binding protein (G protein), q polypeptide |  |
| GNG2 | guanine nucleotide binding protein (G protein), gamma 2 | 10 |
| GNL1 | guanine nucleotide binding protein-like 1 | 8 |
| GRIK3 | glutamate receptor, ionotropic, kainate 3 | 12 |
| GRIK4 | glutamate receptor, ionotropic, kainate 4 | 12 |
| GRIK5 | glutamate receptor, ionotropic, kainate 5 | 12 |
| GRIN2C | glutamate receptor, ionotropic, N-methyl D-aspartate 2C | 12 |
| GRIN2D | glutamate receptor, ionotropic, N-methyl D-aspartate 2D | 12 |
| HPCA | hippocalcin | 7 |
| HTT | huntingtin | 7 |
| KIDINS220 | kinase D-interacting substrate, 220kDa |  |
| LSAMP | limbic system-associated membrane protein | 1 |
| NGF | nerve growth factor (beta polypeptide) |  |
| NINJ1 | ninjurin 1 | 11 |
| NMDA Receptor | -- | 6 |
| Npcd | neuronal pentraxin chromo domain |  |
| NPS | neuropeptide S | 10 |
| NPTXR | neuronal pentraxin receptor | 2 |
| NR3C1 | nuclear receptor subfamily 3, group C, member 1 (glucocorticoid receptor) | 12 |
| NRAS | neuroblastoma RAS viral (v-ras) oncogene homolog |  |
| Nrg1 | neuregulin 1 |  |
| OMG | oligodendrocyte myelin glycoprotein |  |
| OPCML | opioid binding protein/cell adhesion molecule-like | 7 |
| OPRK1 | opioid receptor, kappa 1 | 7 |
| PRND | prion protein 2 (dublet) | 7 |
| PRNP | prion protein | 12 |
| PRODH2 | proline dehydrogenase (oxidase) 2 | 11 |
| PROK2 | prokineticin 2 | 1 |
| PRPH | peripherin | 8 |
| PSMD4 | proteasome (prosome, macropain) 26S subunit, non-ATPase, 4 |  |
| PTK2B | protein tyrosine kinase 2 beta | 9 |
| QRFP | pyroglutamylated RFamide peptide | 5 |
| REST | RE1-silencing transcription factor |  |
| RTN1 | reticulon 1 | 12 |
| SEZ6 | seizure related 6 homolog (mouse) | 1 |
| SHANK2 | SH3 and multiple ankyrin repeat domains 2 |  |
| SLC1A2 | solute carrier family 1 (glial high affinity glutamate transporter), member 2 | 8 |
| SPG11 | spastic paraplegia 11 (autosomal recessive) | 11 |
| SV2B | synaptic vesicle glycoprotein 2B |  |
| TGM6 | transglutaminase 6 |  |
| TH | tyrosine hydroxylase | 11 |
| TNF | tumor necrosis factor |  |
| TRAF3 | TNF receptor-associated factor 3 |  |
| 7S NGF | 7S nerve growth factor | 7 |

**Table S15: list of molecules in networks with basal metabolic functions, oxidative stress and microRNAs associated with genes differentially methylated in frontal cortex and fetal placental tissue**

| **Gene abbreviation** | **Gene name** | **Number of networks** |
| --- | --- | --- |
| ACADS | acyl-CoA dehydrogenase, C-2 to C-3 short chain | 3 |
| ACOT11 | acyl-CoA thioesterase 11 | 7 |
| ADM2 | adrenomedullin 2 |  |
| APOL2 | apolipoprotein L, 2 | 3 |
| DAD1 | defender against cell death 1 | 12 |
| Gcg | glucagon |  |
| GCG | glucagon |  |
| GCLC | glutamate-cysteine ligase, catalytic subunit |  |
| GCLM | glutamate-cysteine ligase, modifier subunit |  |
| GLP1R | glucagon-like peptide 1 receptor |  |
| GLP2R | glucagon-like peptide 2 receptor | 6 |
| Glucocorticoid-GCR | -- | 4 |
| GRB10 | growth factor receptor-bound protein 10 |  |
| GSS | glutathione synthetase | 8 |
| HCRT | hypocretin (orexin) neuropeptide precursor |  |
| HNF4A | hepatocyte nuclear factor 4, alpha | 12 |
| IGFBP2 | insulin-like growth factor binding protein 2, 36kDa | 11 |
| LEP | leptin |  |
| let-7 | microRNA let-7a-1 | 3 |
| MGST2 | microsomal glutathione S-transferase 2 | 9 |
| MIR101 | -- | 21 |
| miR-101b-3p (miRNAs w/seed UACAGUA) | -- |  |
| Mir101c | microRNA 101c | 21 |
| miR-101c (miRNAs w/seed CAGUACU) | -- | 21 |
| mir-124 | microRNA 124-1 | 9 |
| mir-144 | microRNA 144 | 11 |
| mir-17 | microRNA 17 | 17 |
| Mir1936 | microRNA 1936 | 16 |
| miR-1936 (miRNAs w/seed AACUGAC) | -- | 16 |
| Mir1952 | microRNA 1952 | 15 |
| miR-1952 (miRNAs w/seed CUCCACC) | -- | 15 |
| Mir1956 | microRNA mir-1956 | 20 |
| miR-1956 (and other miRNAs w/seed GUCCAGG) | -- | 20 |
| miR-1956-3p (and other miRNAs w/seed GGCUGGC) | -- | 20 |
| mir-218 | microRNA 218-2 | 10 |
| miR-218-5p (and other miRNAs w/seed UGUGCUU) | -- | 10 |
| miR-293-5p (and other miRNAs w/seed CUCAAAC) | -- |  |
| mir-3102 | microRNA 3102 | 22 |
| miR-3102 (and other miRNAs w/seed UCUACUC) | -- | 22 |
| miR-3102-3p (and other miRNAs w/seed AGCACCC) | -- | 22 |
| miR-3102-5p.2-5p (miRNAs w/seed GUGGUGC) | -- | 22 |
| mir-365 | microRNA 365a | 11 |
| miR-365-3p (and other miRNAs w/seed AAUGCCC) | -- | 11 |
| miR-382-5p (miRNAs w/seed AAGUUGU) | -- | 8 |
| miR-4654 (and other miRNAs w/seed GUGGGAU) | -- | 18 |
| miR-489-3p (miRNAs w/seed UGACAUC) | -- |  |
| miR-499-5p (and other miRNAs w/seed UAAGACU) | -- |  |
| miR-515-5p (and other miRNAs w/seed UCUCCAA) | -- |  |
| mir-671 | microRNA 671 | 7 |
| mir-684 | microRNA 684-2 | 14 |
| miR-684 (miRNAs w/seed GUUUUCC) | -- | 14 |
| Mir7004 | microRNA 7004 | 19 |
| miR-7004-3p (miRNAs w/seed CCACUCC) | -- | 19 |
| miR-7004-5p (miRNAs w/seed UCCGUGG) | -- | 19 |
| miR-702-5p (and other miRNAs w/seed UGAGUGG) | -- | 22 |
| Mir7656 | microRNA 7656 | 18 |
| miR-7656-3p (miRNAs w/seed CAGGCUG) | -- | 18 |
| NADK | NAD kinase | 11 |
| NMB | neuromedin B |  |
| NPL | N-acetylneuraminate pyruvate lyase (dihydrodipicolinate synthase) | 5 |
| NR5A2 | nuclear receptor subfamily 5, group A, member 2 |  |
| OGT | O-linked N-acetylglucosamine (GlcNAc) transferase |  |
| OXR1 | oxidation resistance 1 | 7 |
| PARP1 | poly (ADP-ribose) polymerase 1 | 10 |
| PPARG | peroxisome proliferator-activated receptor gamma | 7 |
| PPY | pancreatic polypeptide |  |
| PRKAA1 | protein kinase, AMP-activated, alpha 1 catalytic subunit |  |
| PRKAA2 | protein kinase, AMP-activated, alpha 2 catalytic subunit |  |
| PRKAB2 | protein kinase, AMP-activated, beta 2 non-catalytic subunit |  |
| PRKG1 | protein kinase, cGMP-dependent, type I |  |
| PRKG2 | protein kinase, cGMP-dependent, type II |  |
| RAMP3 | receptor (G protein-coupled) activity modifying protein 3 |  |
| RANBP9 | RAN binding protein 9 |  |
| SLC2A12 | solute carrier family 2 (facilitated glucose transporter), member 12 | 4 |
| SST | somatostatin |  |
| THRB | thyroid hormone receptor, beta |  |
| THRSP | thyroid hormone responsive |  |
| TXN | thioredoxin |  |
| UBC | ubiquitin C | 6,8 |
| VGF | VGF nerve growth factor inducible |  |
| YWHAE | tyrosine 3-monooxygenase/tryptophan 5-monooxygenase activation protein, epsilon | 11 |

**Table S16: Upstream regulators associated with genes differentially methylated in brain and fetal placental tissue**

| **Upstream regulator** | **Molecule type** | **p-value of overlap** |
| --- | --- | --- |
| Stat1-Stat3 | complex | 1.70E-04 |
| FOS | transcription regulator | 2.13E-04 |
| NFIB | transcription regulator | 2.57E-04 |
| ATN1 | transcription regulator | 5.54E-04 |
| miR-382-5p (miRNAs w/seed AAGUUGU) | mature microrna | 5.60E-04 |
| TCL1A | transcription regulator | 5.60E-04 |
| tacrolimus | chemical drug | 6.85E-04 |
| OPRK1 | g-protein coupled receptor | 8.36E-04 |
| olanzapine | chemical drug | 8.59E-04 |
| triamcinolone acetonide | chemical drug | 1.12E-03 |
| Ptk | group | 1.16E-03 |
| magnesium sulfate | chemical drug | 1.54E-03 |
| (+)-MK-801 | chemical drug | 2.07E-03 |
| RANBP9 | other | 2.46E-03 |
| tolbutamide | chemical drug | 2.46E-03 |
| CST3 | other | 2.99E-03 |
| CDK5R1 | kinase | 2.99E-03 |
| clioquinol | chemical drug | 2.99E-03 |
| ryanodine | chemical - endogenous non-mammalian | 2.99E-03 |
| TET1 | other | 3.57E-03 |
| MEIS1 | transcription regulator | 3.57E-03 |
| mannose | chemical - endogenous mammalian | 3.57E-03 |
| HOXC6 | transcription regulator | 3.93E-03 |
| ARHGDIA | other | 4.20E-03 |
| arachidonic acid | chemical - endogenous mammalian | 4.32E-03 |
| PRKAA1 | kinase | 4.68E-03 |
| phorbol esters | chemical - other | 4.68E-03 |
| Srebp | group | 4.87E-03 |
| pyruvic acid | chemical - endogenous mammalian | 4.87E-03 |
| GTF2I | transcription regulator | 5.59E-03 |
| omeprazole | chemical drug | 5.73E-03 |
| progesterone | chemical - endogenous mammalian | 5.99E-03 |
| STK11 | kinase | 6.92E-03 |
| POLR2A | enzyme | 7.16E-03 |
| metyrapone | chemical drug | 7.17E-03 |
| cortivazol | chemical reagent | 7.56E-03 |
| peptide YY 3-36 | biologic drug | 7.56E-03 |
| G protein alpha | group | 7.56E-03 |
| MSK1/2 | group | 7.56E-03 |
| growth factor receptor | group | 7.56E-03 |
| GSK 189254 | chemical drug | 7.56E-03 |
| pridopidine | chemical drug | 7.56E-03 |
| SRF-ELK1 | complex | 7.56E-03 |
| RDH11 | enzyme | 7.56E-03 |
| exendin(9-39) amide | chemical drug | 7.56E-03 |
| FGF14 | growth factor | 7.56E-03 |
| vasopressins | biologic drug | 7.56E-03 |
| ARHGEF17 | other | 7.56E-03 |
| SRSF9 | enzyme | 7.56E-03 |
| KIFAP3 | other | 7.56E-03 |

**Table S17: Genes differentially methylated in both sexes**

| Gene name | Annotation | Chr. | Location | Distance to TSS | Nearest ENSEMBL |
| --- | --- | --- | --- | --- | --- |
| Itgb7 | promoter-TSS (NM_013566) | chr15 | 102062605 | -239 | ENSMUSG00000001281 |
| Tm4sf4 | intron (NM_001168281, intron 3 of 6) | chr3 | 57286074 | 56742 | ENSMUSG00000027801 |
| Gmppa | exon (NM_007463, exon 33 of 41) | chr1 | 75423634 | -8884 | ENSMUSG00000033021 |
| Tiam2 | promoter-TSS (NM_001286757) | chr17 | 3483231 | -259 | ENSMUSG00000023800 |
| Mir671 | promoter-TSS (NR_030423) | chr5 | 24097378 | -554 |  |
| Mtl5 | promoter-TSS (NM_001039658) | chr19 | 3388285 | -572 | ENSMUSG00000024905 |
| Tspo | promoter-TSS (NM_009775) | chr15 | 83393244 | -759 | ENSMUSG00000041736 |
| Mir671 | promoter-TSS (NR_030423) | chr5 | 24097382 | -550 |  |
| Vav2 | promoter-TSS (NM_009500) | chr2 | 27283250 | -905 | ENSMUSG00000009621 |
| C77080 | exon (NM_001033189, exon 6 of 7) | chr4 | 128899433 | 5722 | ENSMUSG00000050390 |
| Tead3 | exon (NM_011566, exon 2 of 13) | chr17 | 28478403 | 8888 | ENSMUSG00000002249 |
| Aldh1a7 | promoter-TSS (NM_011921) | chr19 | 20802740 | -694 | ENSMUSG00000024747 |
| Schip1 | 5' UTR (NM_013928, exon 1 of 7) | chr3 | 68376492 | 202 | ENSMUSG00000027777 |
| Nr3c1 | exon (NM_008173, exon 2 of 8) | chr18 | 39588381 | 58518 | ENSMUSG00000024431 |
| Babam1 | intron (NM_026636, intron 1 of 8) | chr8 | 73921528 | 774 | ENSMUSG00000031820 |
| Gm16853 | exon (NM_010561, exon 19 of 21) | chr9 | 21207822 | 8342 | ENSMUSG00000097665 |
| Dst | exon (NM_001276764, exon 39 of 103) | chr1 | 34245672 | 28497 | ENSMUSG00000026131 |
| Rp1l1 | promoter-TSS (NM_146246) | chr14 | 64610657 | -611 | ENSMUSG00000046049 |
| Plch1 | exon (NM_183191, exon 23 of 23) | chr3 | 63501470 | 153443 | ENSMUSG00000036834 |
| Gna13 | intron (NR_038097, intron 1 of 1) | chr11 | 109222521 | -1587 | ENSMUSG00000020611 |
| Ly86 | promoter-TSS (NM_010745) | chr13 | 37437254 | 40 | ENSMUSG00000021423 |
| Galnt15 | intron (NM_030166, intron 4 of 9) | chr14 | 32859682 | 17393 |  |
| Gm16853 | exon (NM_010561, exon 19 of 21) | chr9 | 21207827 | 8337 | ENSMUSG00000097665 |
| Slc14a2 | promoter-TSS (NM_001110274) | chr18 | 78403681 | -502 | ENSMUSG00000024552 |
| Slc14a2 | promoter-TSS (NM_001110274) | chr18 | 78403707 | -528 | ENSMUSG00000024552 |
| Aldh8a1 | promoter-TSS (NM_178713) | chr10 | 21096536 | -570 | ENSMUSG00000037542 |
| Trim69 | intron (NM_026251, intron 2 of 17) | chr2 | 121984673 | -1763 | ENSMUSG00000033368 |
| Tsc22d1 | promoter-TSS (NM_001177751) | chr14 | 76887484 | -759 | ENSMUSG00000022010 |
| Gsx2 | promoter-TSS (NM_133256) | chr5 | 75471149 | -477 | ENSMUSG00000035946 |
| Tcam1 | exon (NM_029467, exon 7 of 8) | chr11 | 106147790 | 9804 | ENSMUSG00000020712 |
| Asphd2 | promoter-TSS (NM_028386) | chr5 | 112821621 | -388 | ENSMUSG00000029348 |
| Schip1 | L1_Mur3\|LINE\|L1 | chr3 | 68228924 | -43181 | ENSMUSG00000027777 |
| Helz2 | exon (NM_183162, exon 9 of 19) | chr2 | 180969046 | 7686 | ENSMUSG00000027580 |
| Gmppa | exon (NM_007463, exon 30 of 41) | chr1 | 75418309 | -14209 | ENSMUSG00000033021 |
| Brpf3 | exon (NM_001081315, exon 2 of 13) | chr17 | 28944134 | 6063 | ENSMUSG00000063952 |
| Coro2b | exon (NM_175484, exon 2 of 12) | chr9 | 62337117 | 47734 | ENSMUSG00000041729 |
| Tiam2 | promoter-TSS (NM_011878) | chr17 | 3397109 | -98 | ENSMUSG00000023800 |
| Amigo2 | exon (NM_172293, exon 6 of 6) | chr15 | 97214708 | -136990 | ENSMUSG00000048218 |
| Sorbs3 | exon (NM_011366, exon 9 of 21) | chr14 | 70593389 | -1336 | ENSMUSG00000022091 |
| Lrrc34 | intron (NM_026668, intron 3 of 7) | chr3 | 30545829 | 911 | ENSMUSG00000027702 |
| Mst1r | exon (NM_009074, exon 1 of 19) | chr9 | 107810403 | 1183 | ENSMUSG00000032584 |
| Dst | exon (NM_001276764, exon 39 of 103) | chr1 | 34246343 | 29168 | ENSMUSG00000026131 |
| Schip1 | promoter-TSS (NM_001113421) | chr3 | 67868740 | 16 | ENSMUSG00000027777 |
| Asphd2 | exon (NM_028386, exon 1 of 3) | chr5 | 112820615 | 618 | ENSMUSG00000029348 |
| 4933416C03Rik | promoter-TSS (NM_001161855) | chr10 | 115551003 | -30 | ENSMUSG00000074734 |
| Ptgr2 | promoter-TSS (NM_001252626) | chr12 | 85625479 | -767 | ENSMUSG00000072946 |
| Gsx2 | exon (NM_133256, exon 2 of 2) | chr5 | 75472999 | 1373 | ENSMUSG00000035946 |
| Chst5 | promoter-TSS (NM_019950) | chr8 | 114434529 | -430 | ENSMUSG00000031952 |
| Snora47 | exon (NM_028106, exon 2 of 2) | chr13 | 96106133 | 5567 | ENSMUSG00000088108 |
| Zfhx2os | promoter-TSS (NR_004444) | chr14 | 55691223 | -705 | ENSMUSG00000093452 |
| 9530052E02Rik | exon (NM_001081212, exon 1 of 2) | chr8 | 11005631 | -2219 | ENSMUSG00000096938 |
| Tsc22d1 | promoter-TSS (NM_001177751) | chr14 | 76887813 | -430 | ENSMUSG00000022010 |
| Mir3968 | promoter-TSS (NR_039549) | chr11 | 115310198 | -824 | ENSMUSG00000093096 |
| Cacna1d | promoter-TSS (NM_028981) | chr14 | 31304601 | -259 | ENSMUSG00000015968 |
| 1700125H03Rik | exon (NM_028182, exon 6 of 9) | chr8 | 70854988 | -37370 | ENSMUSG00000085197 |
| Dgke | promoter-TSS (NM_019505) | chr11 | 88922836 | -774 | ENSMUSG00000000276 |
| Prpsap2 | promoter-TSS (NM_001164242) | chr11 | 61575976 | -386 | ENSMUSG00000020528 |
| Cul7 | promoter-TSS (NM_025611) | chr17 | 46786731 | -556 | ENSMUSG00000038545 |
| Armcx4 | exon (NM_001202500, exon 6 of 6) | chrX | 131225765 | 4707 | ENSMUSG00000049804 |
| Kcnip4 | promoter-TSS (NM_001199244) | chr5 | 49677579 | -681 | ENSMUSG00000029088 |
| Lpin1 | promoter-TSS (NM_172950) | chr12 | 16597223 | -647 | ENSMUSG00000020593 |
| Gpr12 | exon (NM_008151, exon 1 of 1) | chr5 | 147395134 | 846 | ENSMUSG00000041468 |
| Atxn1 | intron (NM_009124, intron 2 of 7) | chr13 | 46046009 | 14351 | ENSMUSG00000046876 |
| Tns3 | exon (NM_001083587, exon 17 of 31) | chr11 | 8393219 | 171319 | ENSMUSG00000020422 |
| Kcnip4 | intron (NM_001199244, intron 1 of 7) | chr5 | 49096571 | -105650 | ENSMUSG00000029088 |
| Pcdhac2 | exon (NM_001003672, exon 1 of 4) | chr18 | 37306119 | 2496 | ENSMUSG00000007440 |
| Dst | exon (NM_001276764, exon 39 of 103) | chr1 | 34249160 | 31985 | ENSMUSG00000026131 |
| Ccdc162 | promoter-TSS (NM_001177571) | chr10 | 41308312 | -753 | ENSMUSG00000075225 |
| Mir6998 | promoter-TSS (NR_105964) | chr2 | 31467188 | -753 |  |
| Dgke | promoter-TSS (NM_019505) | chr11 | 88922861 | -799 | ENSMUSG00000000276 |
| Icam4 | exon (NM_010493, exon 6 of 7) | chr9 | 20832064 | -1753 | ENSMUSG00000001014 |
| Snord85 | promoter-TSS (NR_028565) | chr4 | 130305351 | -202 |  |
| Tnrc18 | exon (NM_178242, exon 10 of 16) | chr5 | 143533592 | 45474 | ENSMUSG00000039477 |
| Mir467a-4 | promoter-TSS (NR_030470).8 | chr2 | 10397968 | -1 |  |
| Tnks1bp1 | exon (NM_001081260, exon 6 of 12) | chr2 | 84903488 | 12871 | ENSMUSG00000033955 |
| Pdgfrb | exon (NM_008809, exon 3 of 23) | chr18 | 61220895 | 16091 | ENSMUSG00000024620 |
| Ttn | exon (NM_028004, exon 45 of 192) | chr2 | 76746280 | 74324 | ENSMUSG00000051747 |
| Fam105b | intron (NM_001013792, intron 1 of 6) | chr15 | 27558317 | 2131 | ENSMUSG00000046034 |
| Fndc7 | intron (NM_177091, intron 3 of 12) | chr3 | 108684524 | 8402 | ENSMUSG00000045326 |
| Mir8093 | exon (NM_007932, exon 12 of 15) | chr2 | 32534612 | 8674 |  |
| Tnrc18 | exon (NM_178242, exon 10 of 16) | chr5 | 143533780 | 45286 | ENSMUSG00000039477 |
| 4933417O13Rik | promoter-TSS (NR_045842) | chr7 | 150516679 | -124 |  |
| Schip1 | promoter-TSS (NM_001282045) | chr3 | 68271612 | -493 | ENSMUSG00000027777 |
| Gm7120 | promoter-TSS (NM_001039244) | chr13 | 120276493 | -353 | ENSMUSG00000074634 |
| Mir3968 | promoter-TSS (NR_039549) | chr11 | 115309911 | -537 | ENSMUSG00000093096 |
| Htr5a | exon (NM_008314, exon 1 of 2) | chr5 | 28169053 | 566 | ENSMUSG00000039106 |
| Rltpr | exon (NM_001033320, exon 11 of 17) | chr8 | 108219225 | 4419 | ENSMUSG00000050357 |
| Sumo2 | promoter-TSS (NM_133354) | chr11 | 115398391 | -847 | ENSMUSG00000020738 |
| Dst | exon (NM_001276764, exon 39 of 103) | chr1 | 34248311 | 31136 | ENSMUSG00000026131 |
| Slc1a7 | 5' UTR (NM_146255, exon 1 of 11) | chr4 | 107641040 | 101 | ENSMUSG00000008932 |
| Vwf | promoter-TSS (NM_011708) | chr6 | 125502917 | -49 | ENSMUSG00000001930 |
| Hoxd9 | promoter-TSS (NM_013555) | chr2 | 74534939 | -881 | ENSMUSG00000043342 |
| Helz2 | exon (NM_183162, exon 9 of 19) | chr2 | 180969472 | 7260 | ENSMUSG00000027580 |
| Hoxd9 | promoter-TSS (NM_013555) | chr2 | 74535199 | -621 | ENSMUSG00000043342 |
| Col6a4 | promoter-TSS (NM_026763) | chr9 | 105999426 | -404 | ENSMUSG00000032572 |
| Sipa1l2 | promoter-TSS (NM_001081337) | chr8 | 128016693 | -83 | ENSMUSG00000001995 |
| Nav2 | MIRb\|SINE\|MIR | chr7 | 56659212 | 157653 | ENSMUSG00000052512 |
| Zfp957 | exon (NM_001033215, exon 3 of 3) | chr14 | 79613663 | 33511 | ENSMUSG00000071262 |
| Edn2 | promoter-TSS (NM_007902) | chr4 | 119833378 | -651 | ENSMUSG00000028635 |
| Themis3 | exon (NM_008984, exon 29 of 31) | chr17 | 67032774 | -88812 | ENSMUSG00000024105 |
| C77080 | intron (NM_001285867, intron 1 of 6) | chr4 | 128917906 | -1444 | ENSMUSG00000050390 |
| Pcdhgc5 | intron (NM_033582, intron 1 of 3) | chr18 | 37977680 | -1520 | ENSMUSG00000023036 |
| Cacnb1 | exon (NM_031173, exon 2 of 13) | chr11 | 97880999 | -1313 | ENSMUSG00000020882 |
| St3gal3 | exon (NM_172382, exon 12 of 19) | chr4 | 117825860 | -18309 | ENSMUSG00000028538 |
| Anapc2 | intron (NM_175300, intron 3 of 12) | chr2 | 25129618 | 1632 | ENSMUSG00000026965 |
| Crybb3 | promoter-TSS (NM_021352) | chr5 | 113510661 | -57 | ENSMUSG00000029352 |
| 9530052E02Rik | exon (NM_001081212, exon 1 of 2) | chr8 | 11006139 | -1711 | ENSMUSG00000096938 |

**Table S18: s**equences of primers used for pyrosequencing validation of capture sequencing data:

| Gene name | Chr. | CG | Distance to TSS | Nearest ENSEMBL | Sequences of PCR primers |
| --- | --- | --- | --- | --- | --- |
| Olfr322 | 11 | 58478302-58478303 | -761 | ENSMUSG00000063549 | F: TGGGTTTAAGGTAAAGTATGGT R (biot): CACCCTTCCTACCAAAAAAAACTTCA S: GTTTAAGGTAAAGTATGGTT |
| Spp2 | 1 | 90303261-90303262 | -333 | ENSMUSG00000026295 | F: TGGAGGTGGTAGGTAGAGTTAAGTTAATA R (biot): ATACCAAAATCCTCCCTTCCTAA S: AGAGTTAAGTTAATAGGAAAATTAT |
| Tmem56 | 3 | 120966492-120966493 | -259 | ENSMUSG00000028132 | F: TAGGAGGTAAGTGTGTAGAAGAGG R (biot): ATCCAATCCCATTTAAACATCC S: GTATGTTTAAGGTTTTGGAT |
| Tmem56 | 3 | 120967204-120967205 | -971 | ENSMUSG00000028132 | F: GTTAGAAAATTAGTTAGGAAGATTATGTAG R (biot): CCATCACATACCCACACAATTATA S: ATTAGTTAGGAAGATTATGTAGA |
| Tmem56 | 3 | 120967226-120967227 | -993 | ENSMUSG00000028132 | F: GTTAGAAAATTAGTTAGGAAGATTATGTAG R (biot): CCATCACATACCCACACAATTATA S: ATTAGTTAGGAAGATTATGTAGA |
| Trpm1 | 7 | 71413277-71413278 | -18293 | ENSMUSG00000098910 | F: GAGGATGTAAAGTAGTATTTGGATTAGT R (biot): AATCCCATCCAAATCTCAACTCTATACT S: ATTATATTTTTGGTTTTAGTTTAT |
| Tspo | 15 | 83392838-83392839 | -1165 | ENSMUSG00000041736 | F: GGGTTGTAAAGTTTAAGGTTAGAAG R (biot): AATCTAATATCAAAACCAACCAATACA S: ATTTGATAAGAGGGGTT |
| Tspo | 15 | 83393244-83393245 | -759 | ENSMUSG00000041736 | F: GGATTGGTAAGGTTGTAGAGG R (biot): ATCCCCAACTTCCTATTCTTAACCAAC S: TGTTGAGAGATGTTTTAATTAG |
| Morc1 | 16 | 48430184-48430185 | -1165 | ENSMUSG00000022652 | F: TAGATAGAGTGTTTTTTTGGGTTGTAAT R (biot): AATATTTCACATACTCTTCCCAAAATCA S: TTTTTTGGGTTGTAATTTAAATTT |
| Nr3c1 | 18 | 39646956-39646957 | -58 | ENSMUSG00000024431 | F (biot): GGGGAGTTAAGGATTTTTTGGAGT R: CACAAATAACAAATTACAACCTCCATACT S: TTATTTAATAAACTATCTCTTACTA |
