# Supplementary material for: Fetal glucocorticoid receptor (Nr3c1) deficiency alters the landscape of DNA methylation of murine placenta in a sex-dependent manner and is associated to anxiety-like behavior in adulthood
Source: Transl Psychiatry. 2019 Jan 17;9:23. doi: 10.1038/s41398-018-0348-7 (PMC6336883; doi:10.1038/s41398-018-0348-7)
Supplement: Supplementary file 4 — Supplemental Results [file 41398_2018_348_MOESM4_ESM.docx]

**Supplementary results**

**Maternal care behavior**

Maternal behavior of the 19 foster dams is depicted in table S2. All 15 listed behavioral categories appeared with the same statistical probability in each of the 19 foster dams (non-parametric χ^2^−test: χ^2^L/G 323, p=0.241, χ^2^APN 304, p=0.248, χ^2^NB 209, p=0.282, χ^2^ED in nest 170, p=0.279, χ^2^SG in nest 190, p=0.290, χ^2^exploring in nest 152, p=0.308, χ^2^ED out of nest 304, p=0.248, χ^2^SG out of nest 102, p=0.318, χ^2^SR out of nest 18, p=0.324, χ^2^ClDi out of nest 304, p=0.248, χ^2^Stereotypical 36, p=0.287, χ^2^MM 5, p=0.287, χ^2^pups out of nest 5, p=0.287).

**Behavioral tests**

**Locomotor Activity: Novel Cage and Open Field Test**

GR^+/–^ mice showed normal vertical locomotor activity (rearing) in the Novel Cage Test (F_3,44_=0.000, p=0.993) and no significant difference between male and females was found (F_3,44_=0.000, p=0.999). The horizontal locomotor activity and exploratory behavior of GR^+/–^ animals as measured by the Open Field Test also did not differ significantly from control animals: e.g. the total distance moved by GR^+/–^ mice was similar to those of the control group (time*genotype: F_3,44_=0.000, p=1.000; between subject-factor genotype F_3,44=_0.272; p=0.605). Both genotypes moved significantly less in the second half of the test, showing that habituation had taken place as expected (Time: F_3,44_=66.529; p<0.000). However, there was a statistical tendency of female mice moving a greater distance than males (p=0.058, F_3,44_=3.778). Over time, they also show significantly more movement (time*sex p=0.028, F_3,44_=5.194) and more velocity (Time*sex p=0.028, F_3,44_=5.194) than males. For velocity, a significant sex difference could also be found for the between-subjects-factor (p=0.032, F_3,44_=4.879) suggesting more velocity in females.

**Hot Plate Test**

Neither sex (p=0.111, F_3,44_=2.653) nor genotype (p=0.916, F_3.44_=0.011) had a significant influence on pain sensitivity.

**Depressive-like behavior: Forced Swim Test and Learned Helplessness**

Depressive-like behavior as measured by the Forced Swim Test showed that GR^+/–^ mice did not display a significantly different latency to float on day 1 (p=0.285, F_3,44_=1.173) and day2 (p=0.268, F_3,44_=1.253) as well as no significant difference in immobility on both days (total immobility day1: p=0.660, F_3,44_=0.196; total immobility day2: p=0.791, F_3,44_=0.071) compared to wild-types. However, on day 1 immobility time in total was increased in male mice (Sex p=0.004, F_3,44_=9.378), which appeared also in the different time sections of the test in the first two minutes (p=0.013, F_3,44_=6.705), from minute 2 to 4 (Sex p=0.001, F_3,44_=12.328) and still as a tendency from minute 4 to 6 of the test (p=0.058, F_3,44_=3.779).

24 hours later (on day 2), male mice showed a significant increase in immobility from minute 2-4 compared to females (p=0.034, F_3,44_=4.791).

In the Learned Helplessness Paradigm of depression, GR^+/–^ mice displayed a similar escape latency (p=0.668, F_3,41_=0.186) and number of failures to escape (p=0.842, F_3,41_=0.040) in comparison to littermate controls. Yet, male mice had a significantly higher latency to escape (p<0.000, F_3,41_=18.597) and as a tendency, a higher number of failures than females (p=0.084, F_3,41_=3.135). A clear correlation between escape latency and number of failures with male offspring showing the highest values (spearman rho=0.701, p<0.000) was found.

**Body weight, spleen and adrenal size**

GR is crucial for the regulation of metabolic homeostasis in the body. We therefore determined whether GR knockout had an effect on body weight. Body weight steadily increased significantly from birth to the age of 20 weeks (p=0.001, F_3,30_= 6.204). Males were heavier than females (p<0.000, F_3,30_ = 16.832), GR^+/-^ animals weighed the same as wildtype (p=0.487, F_3,30_=0.495). The body weight-adjusted adrenal weight of our sample is significantly higher in females than in males (sex p<0.000, F_3,44_=32.515), the genotype did not cause any difference in adrenal size (p=1.000, F_3,44_=0.000). The same pattern is true for the spleen; females had a significantly larger spleen than males (p<0.000, F_3,43_=48.769), GR^+/-^ did not affect spleen size (p=0.557, F_3,43_=0.350).
