# Supplementary material for: Fetal glucocorticoid receptor (Nr3c1) deficiency alters the landscape of DNA methylation of murine placenta in a sex-dependent manner and is associated to anxiety-like behavior in adulthood
Source: Transl Psychiatry. 2019 Jan 17;9:23. doi: 10.1038/s41398-018-0348-7 (PMC6336883; doi:10.1038/s41398-018-0348-7)
Supplement: Supplementary file 5 — Supplemental legends [file 41398_2018_348_MOESM5_ESM.docx]

**Figure Legends Supplement**

**Figure S1: A.** Pearson’s correlation of all methylation values from capture sequencing with pyro sequencing. **B-F:** Pyro-sequencing validations of capture sequencing arrays of *Olfr322, Tspo, Morc1, Spp2* and *Tmem56*. Each bar plot shows average methylation levels (+/- SEM) of fetal placentae in males and females. Two-factorial (genotype and sex) ANOVA was run and unpaired t test were performed to specify sex*genotype-interactions *p<0.05, **p<0.01, ***p<0.001. Welch’s correction was applied when variances between groups were significantly different.

**Figure S2:** Genome-wide methylation tracks for CpG sites via Integrative Genomics Viewer (broad institute). Comparison is shown for female vs male wildtype, female wildtype vs female *Nr3c1* heterozygous, male wildtype vs male *Nr3c1* heterozygous and male vs female *Nr3c1* heterozygous animals.

**Figures S3: Ingenuity Pathway Analysis:** Canonical pathways for genes hypermethylated in males and hypomethylated in females after Nr3c1 knockout. The first 30 pathways with the lowest p-value are depicted.

**Figure S4: Ingenuity Pathways Analysis:** Canonical pathways for genes hypomethylated in males and hypermethlyated in females after Nr3c1 knockout. The first 30 pathways with the lowest p-value are depicted.
